# Nature Disaster Risk Evaluation with a Group Decision Making Method Based on Incomplete Hesitant Fuzzy Linguistic Preference Relations

**DOI:** 10.3390/ijerph15040751

**Published:** 2018-04-13

**Authors:** Ming Tang, Huchang Liao, Zongmin Li, Zeshui Xu

**Affiliations:** 1 Business School, Sichuan University, Chengdu 610064, China; tangming0716@163.com (M.T.); lizongmin@scu.edu.cn (Z.L.); xuzeshui@263.net (Z.X.); 2 Department of Computer Science and Artificial Intelligence, University of Granada, E-18071 Granada, Spain

**Keywords:** group decision making, incomplete hesitant fuzzy linguistic preference relation, additive consistency, nature disaster risk evaluation

## Abstract

Because the natural disaster system is a very comprehensive and large system, the disaster reduction scheme must rely on risk analysis. Experts’ knowledge and experiences play a critical role in disaster risk assessment. The hesitant fuzzy linguistic preference relation is an effective tool to express experts’ preference information when comparing pairwise alternatives. Owing to the lack of knowledge or a heavy workload, information may be missed in the hesitant fuzzy linguistic preference relation. Thus, an incomplete hesitant fuzzy linguistic preference relation is constructed. In this paper, we firstly discuss some properties of the additive consistent hesitant fuzzy linguistic preference relation. Next, the incomplete hesitant fuzzy linguistic preference relation, the normalized hesitant fuzzy linguistic preference relation, and the acceptable hesitant fuzzy linguistic preference relation are defined. Afterwards, three procedures to estimate the missing information are proposed. The first one deals with the situation in which there are only n−1 known judgments involving all the alternatives; the second one is used to estimate the missing information of the hesitant fuzzy linguistic preference relation with more known judgments; while the third procedure is used to deal with ignorance situations in which there is at least one alternative with totally missing information. Furthermore, an algorithm for group decision making with incomplete hesitant fuzzy linguistic preference relations is given. Finally, we illustrate our model with a case study about flood disaster risk evaluation. A comparative analysis is presented to testify the advantage of our method.

## 1. Introduction

China is one of the few countries with the most serious natural calamities in the world. China’s natural disasters have the following characteristics: the range is wide, the frequency is high, the seasonality is obvious, and the loss is heavy. The formation of natural disasters in China is deeply influenced by natural environment and human activities. The Eastern monsoon area has highly frequent natural disasters and serious losses. China has vast territory, and complex geographical and climatic conditions. In addition to the disasters caused by modern volcanic activity, almost all natural disasters—such as floods, droughts, earthquakes, typhoons, hail, snow disasters, landslides, debris flows, disease and insect pests, and forest fires—occurred every year.

Disaster reduction management must be based on reasonable risk evaluation. In natural disaster risk evaluation, the experiences and knowledge of experts play a critical role. In the process of evaluating, experts would like to use linguistic terms to assess alternatives, which aligns people’s thoughts and speaking manners closely [1]. Rodríguez et al. [2] proposed the Hesitant Fuzzy Linguistic Term Set (HFLTS) to enrich the linguistic elicitation on the basis of fuzzy linguistic approaches and context-free grammars. The HFLTS allows experts to express judgments in more than one linguistic term. For example, the evaluation of a company’s performance can be “*at least good*” and the assessment on a school’s teaching quality can be “*between medium and poor*”. The HFLTS has obtained wide-ranging and deep development since it was proposed [3,4,5,6,7,8,9]. Liao et al. [3] developed a definition of HFLTS in mathematical form and introduced the concept of Hesitant Fuzzy Linguistic Element (HFLE), aiming at making the HFLTS much easier to understand. A comprehensive review on HFLTSs can be found in our previous work [9]. The preference relation is an effective tool in expressing preference information when the experts need to compare alternatives pairwise. Over the past several decades, various kinds of preference relations have been defined, for instance, the fuzzy preference relation [10], the multiplicative preference relation [11], and the hesitant fuzzy preference relation [12]. Rodríguez et al. [6] initially proposed the definition of hesitant fuzzy linguistic preference relation (HFLPR). Liu et al. [13] enhanced the definition of HFLPR into a subscript-symmetric form. Zhu and Xu [14] further proposed the definition of HFLPR in a mathematical form. The HFLPR is a powerful tool to represent the situations in which the experts are hesitant among a set of probable linguistic values for the preference degrees of pairwise comparisons on alternatives.

It is noted that a complete preference matrix has n(n−1)/2 judgments, which means that an expert needs to make n(n−1)/2 comparisons. When n is large, the expert’s workload is heavy. Furthermore, sometimes an expert does not have the ability to discriminate the degree to which an alternative is better than another. While we cannot force the experts to provide their opinions as this may produce the wrong information, it is necessary for us to provide tools, allowing the experts to express the lack of knowledge. As a result, an incomplete HFLPR (IHFLPR) with missing information is constructed. However, there is little research focusing on the study of estimating the missing information in an HFLPR. In the environment of IHFLPR, Ren et al. [15] established two models that consider the satisfaction degrees of experts and applied several experiments in evaluating these models. Then, the models introduced were used in site selection for a hydropower station. However, they did not propose the approaches to estimate the missing information. In this paper, we will study this issue.

To do this, in our paper, some theoretical findings related to IHFLPRs are investigated in detail. The novelties of this paper are highlighted as follows:

Some characteristics of an additive consistent HFLPR are studied. Two theorems to facilitate further study on estimating the missing information of a HFLPR are given. To deal with the situation in which the calculation results over the HFLEs do not fall in the original Linguistic Term Set (LTS), a linear function to unify the terms within the given LTS is proposed.

The definition of the IHFLPR, the normalized IHFLPR and the acceptable IHFLPR are initially introduced. The IHFLPRs are divided into three categories: the IHFLPR with n−1 known judgments (one particular alternative is compared with all other alternatives only once), the IHFLPR with more than n−1 judgments, and the IHFLPR with one alternative with totally missing information. We put forward three approaches to estimate the missing values of IHFLPRs with the above three categories.

A novel method to measure the distance between two HFLTSs is proposed. Based on which, an algorithm for group decision making with IHFLPRs is established.

A real-world case study about flood disaster risk evaluation is provided to verify the feasibility of the Group Decision Making (GDM) model.

The rest of this paper is organized as follows: In Section 2, we briefly review the concepts of HFLTSs and HFLPRs. Some characteristics related to the additive consistent HFLPR are discussed in Section 3. In Section 4, the definition of the IHFLPR and three proposed approaches to construct a complete HFLPR are introduced. An algorithm for GDM with the IHFLPRs is given in Section 5. Section 6 illustrates an example in terms of flood disaster risk assessment. Section 7 concludes this paper with some remarks.

## 2. Preliminaries

In this section, we briefly review the HFLTS and the HFLPR.

### 2.1. HFLTS

In many real-world decision situations, experts would like to use some possible linguistic terms to assess alternatives. To represent these problems, Rodríguez et al. [2] introduced the concept of HFLTS.

**Definition** **1.** [2] *Let* $\bar{S}=\{s_{0},s_{1},\ldots ,s_{g}\}$ *be a LTS. A HFLTS,* $h_{\bar{S}}$*, is an ordered finite subset of the consecutive linguistic terms of* $\bar{S}$.

In Definition 1, the subscripts of S are asymmetric. This kind of definition may cause some problems [4]. To overcome this limitation, Liao et al. [3] developed the mathematical definition of HFLTS and used the subscript-symmetric LTS.

**Definition** **2.** [3] *Let $S=\{s_{\alpha }|\alpha \in [-\tau ,\tau ]\}$ be a LTS. A HFLTS on X, $B_{S}$, is in mathematical terms of*

(1) $$B_{S}=\left\{\langle x,b_{S}(x)\rangle |x\in X\right\}$$

*where $b_{S}(x)=\{s_{\phi_{l}}(x)|s_{\phi_{l}}(x)\in S,l=1,2,\ldots ,L\}$ with L being the number of linguistic terms in $b_{S}(x)$. We call $b_{S}(x)$ the hesitant fuzzy linguistic element (HFLE)*.

In the following section of this paper, we use the subscript-symmetric linguistic term set S for our discussion. For example, let $S=\{s_{-4}=extremely\;low,\;s_{-3}=very\;low,$ $s_{-2}=low,\;s_{-1}=slightly\;low,$ $s_{0}=medium,$ $s_{1}=slightly\;high,$ $s_{2}=high,\;s_{3}=very\;high,\;s_{4}=extremely\;high\}$ be a LTS. Based on S, we give two HFLTSs as $b_{1}=\{s_{-3}=very\;low,\;s_{-2}=low\}$, $b_{2}=\{s_{1}=slightly\;high,\;s_{2}=high,\;s_{-3}=very\;high\}$, or briefly, $b_{1}=\{s_{-3},s_{-2}\}$, $b_{2}=\{s_{1},s_{2},s_{3}\}$.

Linguistic expressions align closely with people’s thoughts and manner of speaking. Rodríguez et al. [2] developed a context-free grammar and a function to transform the linguistic expressions into HFLEs.

Given a HFLE b, $s(b)=\sum_{s_{\alpha }\in \beta }\left(\frac{\alpha }{2\tau }\right)/l_{b}$ is called the score function of b, where $l_{b}$ is the number of linguistic terms in b [5]. For two HFLEs $b_{1}$ and $b_{2}$, if $s(b_{1})>s(b_{2})$, then $b_{1}>b_{2}$; if $s(b_{1})=s(b_{2})$, we have $b_{1}=b_{2}$.

To aggregate the HFLEs, Zhang and Wu [5] proposed the hesitant fuzzy linguistic weighted averaging (HFLWA) operator:

**Definition** **3.** [5] *Let $b_{i}(i=1,2,\ldots ,n)$ be a collection of HFLTSs. A HFLWA operator is defined as*

(2) $$\mathrm{HFLWA}(b_{1},b_{2},\ldots ,b_{n})=\overset{n}{\underset{i=1}{⊕}}\left(\frac{1}{n}b_{i}\right)=\underset{s_{\alpha_{1}}\in b_{1},s_{\alpha_{2}}\in b_{2},s_{\alpha_{3}}\in b_{3}}{\cup }\left\{s_{\sum_{i=1}^{n}\frac{\alpha_{i}}{n}}\right\}$$

Let I:S→[−τ,τ] be a function to transform the linguistic term to its subscript, such that

(3) $$I(s_{\alpha })=\alpha$$

### 2.2. HFLPR

When expressing preference information over alternatives, experts often give pairwise comparisons of alternatives by linguistic expressions. Based on context-free grammars, Rodríguez et al. [6] introduced a model which can use several linguistic terms to express the pairwise preferences on alternatives. It is noted that the HFLPR defined by Rodríguez et al. [6] is asymmetric. After that, Liu et al. [13] extended the definition of HFLPR in which the elements $b_{ij}(i,j=1,2,\ldots ,n)$ have the following forms: (1) the single term $s_{l}\in S$; (2) the expression “*at least* $s_{l}$”, $s_{l}\in S$; (3) The expression “*at most* $s_{l}$”, $s_{l}\in S$; and (4) the expression “*between* $s_{l}$ *and* $s_{m}$”, where $s_{l}<s_{m}$, $s_{l}\in S$. The HFLPR proposed by Liu et al. [13] satisfied the following conditions: (1) $b_{ii}=s_{0}$; (2) if $b_{ij}=s_{l}$, then $b_{ji}=s_{-l}$; (3) if $b_{ji}$ = “*at least* $s_{l}$”, then $b_{ji}$ = “*at most* $s_{l}$”; (4) if $b_{ji}$= “*at most* $s_{l}$”, then $b_{ji}$ = “*at least* $s_{-l}$”; and (5) if $b_{ij}$ = “*between* $s_{l}$ *and* $s_{m}$”, then $b_{ji}$ = “*between* $s_{-m}$ *and* $s_{-l}$”.

Motivated by the above results, Zhu and Xu [14] introduced the mathematical definition of HFLPR:

**Definition** **4.** [14] *A HFLPR $B={\left(b_{ij}\right)}_{n\times n}\subset X\times X$, where $b_{ij}=\{b_{ij}^{\sigma (l)}|l=1,2,\ldots ,L_{b_{ij}}\}$ ($L_{b_{ij}}$ is the number of linguistic terms in $b_{ij}$) is an HFLE, denoting the hesitant degrees to which $x_{i}$ is preferred to $x_{j}$. For all i,j=1,2,…,n, $b_{ij}(i<j)$ should satisfy the conditions as follows:*

(4) $$b_{ij}^{\sigma (l)}⊕b_{ji}^{\sigma (l)}=s_{0},\;b_{ii}=s_{0},\;L_{b_{ij}}=L_{b_{ji}},\;b_{ij}^{\sigma (l)}<b_{ij}^{\sigma (l+1)},\;b_{ji}^{\sigma (l+1)}<b_{ji}^{\sigma (l)}$$

*where $b_{ij}^{\sigma (l)}$ and $b_{ji}^{\sigma (l)}$ are the the lth elements in $b_{ij}$ and $b_{ji}$, respectively*.

**Example** **1.** *Assume that an expert assesses four alternatives and constructs a HFLPR as:*

$$B=\left\{\begin{array}{cccc} \left\{s_{0}\right\} & \left\{s_{-2},s_{-1}\right\} & \left\{s_{2},s_{3},s_{4}\right\} & \left\{s_{-3},s_{-2},s_{-1}\right\}\\ \left\{s_{2},s_{1}\right\} & \left\{s_{0}\right\} & \left\{s_{1}\right\} & \left\{s_{4}\right\}\\ \left\{s_{-2},s_{-3},s_{-4}\right\} & \left\{s_{-1}\right\} & \left\{s_{0}\right\} & \left\{s_{0},s_{1}\right\}\\ \left\{s_{3},s_{2},s_{1}\right\} & \left\{s_{-4}\right\} & \left\{s_{0},s_{-1}\right\} & \left\{s_{0}\right\} \end{array}\right\}$$

It is observed that the experts usually provide different numbers of linguistic terms when comparing alternatives in pairwise, for instance, in Example 1, $b_{12}=\{s_{-2},s_{-1}\}$ and $b_{13}=\{s_{2},s_{3},s_{4}\}$. To facilitate the calculation on HFLEs, Zhu and Xu [14] developed two normalization principles to make different HFLEs have the same number of linguistic terms:

(1) α-normalization: Remove some elements of $b_{i}$ whose number of elements is more than others; (2) β-normalization: Add some elements of $b_{i}$ whose number of elements is less than others. Let $b^{+}$ and $b^{-}$ be the max and min linguistic terms of the HFLE $b=\{b^{\sigma (l)}|l=1,2,\ldots ,L_{b}\}$, respectively, and δ(0≤δ≤1) be an optimized parameter. With the β-normalization principle, $\bar{b}=\delta b^{+}+(1-\delta )b^{-}$ can be taken as the added linguistic term. Once all the HFLEs in a HFLPR are extended to the same length, a normalized HFLPR can be established.

**Definition** **5.** [14] *For a HFLPR $B={\left(b_{ij}\right)}_{n\times n}$, we use the optimized parameter δ(0≤δ≤1) to add linguistic terms to $b_{ij}$ (i<j) and use the parameter 1−δ to add linguistic terms to $b_{ji}$ (i<j). We can obtain a normalized HFLPR $\bar{B}={\left({\bar{b}}_{ij}\right)}_{n\times n}$, satisfying*

(5) $$L_{{\bar{b}}_{ij}}=\max\{L_{b_{ij}}|i,j=1,2,\ldots ,n,i\neq j\}$$

*where $L_{{\bar{b}}_{ij}}$ is the number of linguistic terms in ${\bar{b}}_{ij}$*.

**Example** **2.** *(Continued to Example 1) For the HFLPR in Example 1, let δ=1, the normalized HFLPR $\bar{B}$ can be obtained as:*

$$\bar{B}=\left\{\begin{array}{cccc} \left\{s_{0}\right\} & \left\{s_{-2},s_{-1},s_{-1}\right\} & \left\{s_{2},s_{3},s_{4}\right\} & \left\{s_{-3},s_{-2},s_{-1}\right\}\\ \left\{s_{2},s_{1},s_{1}\right\} & \left\{s_{0}\right\} & \left\{s_{1},s_{1},s_{1}\right\} & \left\{s_{4},s_{4},s_{4}\right\}\\ \left\{s_{-2},s_{-3},s_{-4}\right\} & \left\{s_{-1},s_{-1},s_{-1}\right\} & \left\{s_{0}\right\} & \left\{s_{0},s_{1},s_{1}\right\}\\ \left\{s_{3},s_{2},s_{1}\right\} & \left\{s_{-4},s_{-4},s_{-4}\right\} & \left\{s_{0},s_{-1},s_{-1}\right\} & \left\{s_{0}\right\} \end{array}\right\}$$

The HFLPR has been studied in various aspects, including the consistency measures of the HFLPR [14,15,16,17,18], the priority derivation from the HFLPR [19], and the consensus reaching process with HFLPRs [19,20]. After transforming the HFLPR into its corresponding normalized HFLPR by the β-normalization principle, Zhu and Xu [14] discussed the additive consistency of the HFLPR. Based on the possibility distribution-based method, Wu and Xu [16] further gave the definition of the additive consistency of the HFLPR and proposed a consistency improving method. To overcome the limitation of the normalized process in previous work [14], based on graph theory, Wang and Xu [17] introduced a new additive consistency of the extended HFLPR. In addition, Zhang and Wu [18] defined the multiplicative consistency of the HFLPR. In view of the statistical distributions, Wang and Gong [19] established chance-restricted programming to derive the priority weights from the HFLPR. As for the consensus reaching process of HFLPRs, Dong et al. [20] proposed a consensus measure based on the distance to the preference relations and then advanced an optimization-based consensus model for GDM with HFLPRs. Wu and Xu [21] gave an interactive consensus model based on the distance to the collective preference.

## 3. Properties of the Additive Consistent HFLPR

In this section, we study the properties of the additive consistent HFLPR.

Based on Definition 5, Zhu and Xu [14] introduced the concept of additive consistent HFLPR.

**Definition** **6.** [14] *Given a HFLPR $B={\left(b_{ij}\right)}_{n\times n}$ and its normalized HFLPR $\bar{B}={\left({\bar{b}}_{ij}\right)}_{n\times n}$ with δ, if*

(6) $${\bar{b}}_{ij}^{\sigma (l)}={\bar{b}}_{ik}^{\sigma (l)}⊕{\bar{b}}_{kj}^{\sigma (l)}\;\left(i,j,k=1,2,\ldots ,n\right)$$

*then B is an additive consistent HFLPR*.

**Theorem** **1.** *For a HFLPR $B={\left(b_{ij}\right)}_{n\times n}$ and its corresponding normalized HFLPR $\bar{B}={\left({\bar{b}}_{ij}\right)}_{n\times n}$ with δ, the following statements are equivalent:*

(a) $\bar{B}$ *is additive consistent;*
(b) $I\left({\bar{b}}_{i(i+1)}^{\sigma (l)}\right)+I\left({\bar{b}}_{\left(i+1)(i+2\right)}^{\sigma (l)}\right)+\cdots +I\left({\bar{b}}_{(j-1)j}^{\sigma (l)}\right)+I\left({\bar{b}}_{ji}^{\sigma (l)}\right)=0$, ∀i<j;
(c) $I\left({\bar{b}}_{ij_{1}}^{\sigma (l)}\right)+I\left({\bar{b}}_{j_{1}j_{2}}^{\sigma (l)}\right)+\cdots +I\left({\bar{b}}_{j_{t-1}j_{t}}^{\sigma (l)}\right)+I\left({\bar{b}}_{j_{t}i}^{\sigma (l)}\right)=0$, $\forall i<j_{1}<j_{2}<\cdots <j_{t}$.

**Proof.**  (a) ⇒ (b) Let i<j, k=j−i. The expression (a) can be rewritten in a mathematical form: $I\left({\bar{b}}_{ij}^{\sigma (l)}\right)=I\left({\bar{b}}_{ik}^{\sigma (l)}\right)+I\left({\bar{b}}_{kj}^{\sigma (l)}\right)$ (i,j,k=1,2,…,n). ${\bar{b}}_{ij}$ satisfies $I\left({\bar{b}}_{ij}^{\sigma (l)}\right)+I\left({\bar{b}}_{ji}^{\sigma (l)}\right)=0$, $I\left({\bar{b}}_{ii}^{\sigma (l)}\right)=0$.

Mathematical induction is used to prove this part. When k=j−i=1, it is obvious. Next we assume that the property is true for k=n, that is, $I\left({\bar{b}}_{i(i+1)}^{\sigma (l)}\right)+I\left({\bar{b}}_{\left(i+1)(i+2\right)}^{\sigma (l)}\right)+\cdots I\left({\bar{b}}_{\left(i+n-1)(i+n\right)}^{\sigma (l)}\right)+I\left({\bar{b}}_{(i+n)i}^{\sigma (l)}\right)=0$. Then for k=n+1, we have

$$I\left({\bar{b}}_{i(i+1)}^{\sigma (l)}\right)+I\left({\bar{b}}_{\left(i+1)(i+2\right)}^{\sigma (l)}\right)+\cdots +I\left({\bar{b}}_{\left(i+n-1)(i+n\right)}^{\sigma (l)}\right)+I\left({\bar{b}}_{\left(i+n)(i+n+1\right)}^{\sigma (l)}\right)+I\left({\bar{b}}_{(i+n+1)i}^{\sigma (l)}\right)\;=\left(I\left({\bar{b}}_{i(i+1)}^{\sigma (l)}\right)+I\left({\bar{b}}_{\left(i+1)(i+2\right)}^{\sigma (l)}\right)+\cdots +I\left({\bar{b}}_{\left(i+n-1)(i+n\right)}^{\sigma (l)}\right)\right)+I\left({\bar{b}}_{\left(i+n)(i+n+1\right)}^{\sigma (l)}\right)+I\left({\bar{b}}_{(i+n+1)i}^{\sigma (l)}\right)\;=-I\left({\bar{b}}_{(i+n)i}^{\sigma (l)}\right)+I\left({\bar{b}}_{\left(i+n)(i+n+1\right)}^{\sigma (l)}\right)+I\left({\bar{b}}_{(i+n+1)i}^{\sigma (l)}\right)=I\left({\bar{b}}_{i(i+n)}^{\sigma (l)}\right)+I\left({\bar{b}}_{\left(i+n)(i+n+1\right)}^{\sigma (l)}\right)+I\left({\bar{b}}_{(i+n+1)i}^{\sigma (l)}\right)=0$$

Therefore, the expression (b) is confirmed.

(b) ⇒ (a) Based on the condition, we have

$$\begin{array}{c} I\left({\bar{b}}_{ij}^{\sigma (l)}\right)+I\left({\bar{b}}_{jk}^{\sigma (l)}\right)+I\left({\bar{b}}_{ki}^{\sigma (l)}\right)=-I\left({\bar{b}}_{ji}^{\sigma (l)}\right)-I\left({\bar{b}}_{kj}^{\sigma (l)}\right)+I\left({\bar{b}}_{ki}^{\sigma (l)}\right)\\ =\left(I\left({\bar{b}}_{i(i+1)}^{\sigma (l)}\right)+I\left({\bar{b}}_{\left(i+1)(i+2\right)}^{\sigma (l)}\right)+\cdots +I\left({\bar{b}}_{(j-1)j}^{\sigma (l)}\right)\right)+\left(I\left({\bar{b}}_{j(j+1)}^{\sigma (l)}\right)+I\left({\bar{b}}_{\left(j+1)(j+2\right)}^{\sigma (l)}\right)+\cdots +I\left({\bar{b}}_{(k-1)k}^{\sigma (l)}\right)\right)+I\left({\bar{b}}_{ki}^{\sigma (l)}\right)\\ =\left(I\left({\bar{b}}_{i(i+1)}^{\sigma (l)}\right)+I\left({\bar{b}}_{\left(i+1)(i+2\right)}^{\sigma (l)}\right)+\cdots +I\left({\bar{b}}_{(j-1)j}^{\sigma (l)}\right)+I\left({\bar{b}}_{j(j+1)}^{\sigma (l)}\right)+I\left({\bar{b}}_{\left(j+1)(j+2\right)}^{\sigma (l)}\right)+\cdots +I\left({\bar{b}}_{(k-1)k}^{\sigma (l)}\right)+I\left({\bar{b}}_{ki}^{\sigma (l)}\right)\right)=0. \end{array}$$

Thus, the expression (a) is confirmed.

(a) ⇒ (c) Similar to the proof of (a) ⇒ (b), mathematical induction is used to prove this part. When t=1, the expression (c) is given as $I\left({\bar{b}}_{ij_{1}}^{\sigma (l)}\right)+I\left({\bar{b}}_{j_{1}i}^{\sigma (l)}\right)=0$. It is clearly true.

Next, we assume that the hypothesis is true when t=n, i.e., $I\left({\bar{b}}_{ij_{1}}^{\sigma (l)}\right)+I\left({\bar{b}}_{j_{1}j_{2}}^{\sigma (l)}\right)+\cdots +I\left({\bar{b}}_{j_{n-1}j_{n}}^{\sigma (l)}\right)+I\left({\bar{b}}_{j_{n}i}^{\sigma (l)}\right)=0$, $\forall i<j_{1}<j_{2}<\cdots <j_{n}$. Then for t=n+1, we have $I\left({\bar{b}}_{ij_{1}}^{\sigma (l)}\right)+I\left({\bar{b}}_{j_{1}j_{2}}^{\sigma (l)}\right)+\cdots +I\left({\bar{b}}_{j_{n-1}j_{n}}^{\sigma (l)}\right)+I\left({\bar{b}}_{j_{n}j_{n+1}}^{\sigma (l)}\right)+I\left({\bar{b}}_{j_{n+1}i}^{\sigma (l)}\right)=\left(I\left({\bar{b}}_{ij_{1}}^{\sigma (l)}\right)+I\left({\bar{b}}_{j_{1}j_{2}}^{\sigma (l)}\right)+\cdots +I\left({\bar{b}}_{j_{n-1}j_{n}}^{\sigma (l)}\right)\right)+I\left({\bar{b}}_{j_{n}j_{n+1}}^{\sigma (l)}\right)+I\left({\bar{b}}_{j_{n+1}i}^{\sigma (l)}\right)=-I\left({\bar{b}}_{j_{n}i}^{\sigma (l)}\right)+I\left({\bar{b}}_{j_{n}j_{n+1}}^{\sigma (l)}\right)+I\left({\bar{b}}_{j_{n+1}i}^{\sigma (l)}\right)=I\left({\bar{b}}_{ij_{n}}^{\sigma (l)}\right)+I\left({\bar{b}}_{j_{n}j_{n+1}}^{\sigma (l)}\right)+I\left({\bar{b}}_{j_{n+1}i}^{\sigma (l)}\right)=0$.

(c) ⇒ (a) Based on the condition, we have $I\left({\bar{b}}_{ij}^{\sigma (l)}\right)+I\left({\bar{b}}_{jk}^{\sigma (l)}\right)+I\left({\bar{b}}_{ki}^{\sigma (l)}\right)=-I\left({\bar{b}}_{ji}^{\sigma (l)}\right)-I\left({\bar{b}}_{kj}^{\sigma (l)}\right)+I\left({\bar{b}}_{ki}^{\sigma (l)}\right)=\left(I\left({\bar{b}}_{ij_{1}}^{\sigma (l)}\right)+I\left({\bar{b}}_{j_{1}j_{2}}^{\sigma (l)}\right)+\cdots +I\left({\bar{b}}_{j_{t-1}j_{}}^{\sigma (l)}\right)\right)+\left(I\left({\bar{b}}_{jk_{1}}^{\sigma (l)}\right)+I\left({\bar{b}}_{k_{1}k_{2}}^{\sigma (l)}\right)+\cdots +I\left({\bar{b}}_{k_{t-1}k}^{\sigma (l)}\right)\right)+I\left({\bar{b}}_{ki}^{\sigma (l)}\right)=I\left({\bar{b}}_{ij_{1}}^{\sigma (l)}\right)+I\left({\bar{b}}_{j_{1}j_{2}}^{\sigma (l)}\right)+\cdots +I\left({\bar{b}}_{j_{t-1}j_{}}^{\sigma (l)}\right)+I\left({\bar{b}}_{jk_{1}}^{\sigma (l)}\right)+I\left({\bar{b}}_{k_{1}k_{2}}^{\sigma (l)}\right)+\cdots +I\left({\bar{b}}_{k_{t-1}k}^{\sigma (l)}\right)+I\left({\bar{b}}_{ki}^{\sigma (l)}\right)=0$. This completes the proof of Theorem 1. ☐

**Note**. The values in Theorem 1(b) are arranged in consecutive orders, for instance, $I\left({\bar{b}}_{12}^{\sigma (l)}\right)$, $I\left({\bar{b}}_{23}^{\sigma (l)}\right)$, $I\left({\bar{b}}_{34}^{\sigma (l)}\right)$ and $I\left({\bar{b}}_{41}^{\sigma (l)}\right)$, while the values in Theorem 1(c) are not necessarily arranged in consecutive orders, for instance, $I\left({\bar{b}}_{13}^{\sigma (l)}\right)$, $I\left({\bar{b}}_{35}^{\sigma (l)}\right)$, $I\left({\bar{b}}_{58}^{\sigma (l)}\right)$ and $I\left({\bar{b}}_{81}^{\sigma (l)}\right)$. Thus, Theorem 1(b) is a special case of Theorem 1(c). Theorem 1 reveals an important character of the additive consistent HFLPR. It plays a key role in estimating the missing information of incomplete HFLPR.

**Theorem** **2.** *Let $B={\left(b_{ij}\right)}_{n\times n}$ be a HFLPR and $\bar{B}={\left({\bar{b}}_{ij}\right)}_{n\times n}$ be its associated normalized HFLPR with δ. Then B is additive consistent if and only if*

(7) $$I\left({\bar{b}}_{ij}^{\sigma (l)}\right)-I\left({\bar{b}}_{ik}^{\sigma (l)}\right)=I\left({\bar{b}}_{lj}^{\sigma (l)}\right)-I\left({\bar{b}}_{lk}^{\sigma (l)}\right),\;\forall i,p\leq j<k$$

**Proof.** 

(1) **Sufficiency**. If $I\left({\bar{b}}_{ij}^{\sigma (l)}\right)-I\left({\bar{b}}_{ik}^{\sigma (l)}\right)=I\left({\bar{b}}_{pj}^{\sigma (l)}\right)-I\left({\bar{b}}_{pk}^{\sigma (l)}\right)$, ∀i,p≤j<k. Let p=j. Since $I\left({\bar{b}}_{jj}^{\sigma (l)}\right)$ = 0, we have $I\left({\bar{b}}_{ij}^{\sigma (l)}\right)-I\left({\bar{b}}_{ik}^{\sigma (l)}\right)=0-I\left({\bar{b}}_{jk}^{\sigma (l)}\right)$, ∀i≤j<k. That is, $I\left({\bar{b}}_{ij}^{\sigma (l)}\right)=I\left({\bar{b}}_{jk}^{\sigma (l)}\right)+I\left({\bar{b}}_{ki}^{\sigma (l)}\right)$, ∀i≤j<k. Therefore, B is additive consistent.
(2) **Necessity**. If B is additive consistent, we can get $I\left({\bar{b}}_{ij}^{\sigma (l)}\right)=I\left({\bar{b}}_{jk}^{\sigma (l)}\right)+I\left({\bar{b}}_{ki}^{\sigma (l)}\right)$ and $I\left({\bar{b}}_{ij}^{\sigma (l)}\right)-I\left({\bar{b}}_{ik}^{\sigma (l)}\right)=-I\left({\bar{b}}_{jk}^{\sigma (l)}\right)$, ∀i≤j<k. Similarly, $I\left({\bar{b}}_{lj}^{\sigma (l)}\right)=I\left({\bar{b}}_{lk}^{\sigma (l)}\right)+I\left({\bar{b}}_{kl}^{\sigma (l)}\right)$ and $I\left({\bar{b}}_{lj}^{\sigma (l)}\right)-I\left({\bar{b}}_{lk}^{\sigma (l)}\right)=-I\left({\bar{b}}_{jk}^{\sigma (l)}\right)$, ∀l≤j<k. Thus, $I\left({\bar{b}}_{ij}^{\sigma (l)}\right)-I\left({\bar{b}}_{ik}^{\sigma (l)}\right)=I\left({\bar{b}}_{lj}^{\sigma (l)}\right)-I\left({\bar{b}}_{lk}^{\sigma (l)}\right)$, ∀i,p≤j<k. This completes the proof of Theorem 2. ☐

From Theorem 2, another important property of the additive consistent HFLPR can be obtained. That is, the difference between the elements in two columns is a constant value for all rows.

**Note**. It is noted that, in the calculation process, some virtual linguistic terms that are not in the original LTS $\left\{s_{\alpha }|\alpha \in [-\tau ,\tau ]\right\}$ may be obtained. For example, let τ=4, ${\bar{b}}_{12}^{\sigma (1)}=s_{2}$, ${\bar{b}}_{23}^{\sigma (1)}=s_{3}$. Then, we have $I\left({\bar{b}}_{12}^{\sigma (1)}\right)+I\left({\bar{b}}_{23}^{\sigma (1)}\right)=5$. $s_{5}\notin \{s_{\alpha }|\alpha \in [-4,4]\}$. In this situation, a matrix $\bar{B}={\left({\bar{b}}_{ij}\right)}_{n\times n}$ with entries in $\left\{s_{\alpha }|\alpha \in [-a-\tau ,a+\tau ]\right\}$ where $s_{-a-\tau }$ is the worst linguistic term in the matrix $\bar{B}$ and $s_{a+\tau }$ is the best linguistic term in the matrix $\bar{B}$ is obtained. We need a conversion function F to make the virtual linguistic terms be in $\left\{s_{\alpha }|\alpha \in [-\tau ,\tau ]\right\}$, where F:[−a−τ,a+τ]→[−τ,τ], satisfying (a) F(−a−τ)=−τ; (b) F(a+τ)=τ. Then, a transformation function that meets the conditions (a) and (b) is used to make the virtual linguistic terms fall in $\left\{s_{\alpha }|\alpha \in [-\tau ,\tau ]\right\}$:

(8) $$F(x)=\frac{\tau x}{\tau +a}$$

## 4. Incomplete HFLPR and Some Repairing Procedures for Inconsistent IHFLPR

In this section, the definition of the IHFLPR is introduced. The IHFLPRs are divided into three categories: the IHFLPR with n−1 judgments (one particular alternative is compared with all other alternatives only once); the IHFLPR with more than n−1 judgments; the IHFLPR with one alternative having no information. Three approaches to induce these three kinds of IHFLPRs to the complete HFLPRs are put forward.

### 4.1. The Incomplete HFLPRs

A complete HFLPR regarding to n alternatives has n×(n−1)/2 judgments that need to be determined. However, when n is large, the workload is heavy. Furthermore, the experts often lack knowledge for a problem, which means they cannot provide complete information. As a result, the IHFLPR with missing elements is constructed. The IHFLPR in mathematical form is defined as below:

**Definition** **7.** *Let $B={\left(b_{ij}\right)}_{n\times n}$ be a HFLPR, where $b_{ij}=\{b_{ij}^{\sigma (l)}|s=1,2,\ldots ,L_{b_{ij}}\}(i,j=1,2,\ldots ,n)$. If some elements cannot be given by the expert, then we call B an IHFLPR. We use “x” to represent the missing elements. The known elements provided by the expert satisfy*

(9) $$b_{ij}^{\sigma (l)}⊕b_{ji}^{\sigma (l)}=s_{0},\;b_{ii}=s_{0},\;L_{b_{ij}}=L_{b_{ji}},\;b_{ij}^{\sigma (l)}<b_{ij}^{\sigma (l+1)},\;b_{ji}^{\sigma (l+1)}<b_{ji}^{\sigma (l)},\;\forall b_{ij}\in \Phi$$

*where Φ is the set of all known elements in B*.

The missing elements can be resulted from the incapacity or inexperience of an expert who cannot provide clear view on how the alternative $x_{i}$ is better. For example, an IHFLPR can be given as

$$B=\left\{\begin{array}{cccc} \left\{s_{0}\right\} & x & \left\{s_{2},s_{3}\right\} & x\\ x & \left\{s_{0}\right\} & x & \left\{s_{-4},s_{-3},s_{-2}\right\}\\ \left\{s_{-2},s_{-3}\right\} & x & \left\{s_{0}\right\} & \left\{s_{4}\right\}\\ x & \left\{s_{4},s_{3},s_{2}\right\} & \left\{s_{-4}\right\} & \left\{s_{0}\right\} \end{array}\right\}$$

It should be noted that the HFLEs of a HFLPR often have different numbers of linguistic terms. Therefore, a normalization process is necessary. Based on Definition 5, the β-normalization principle is used to add linguistic terms and obtain a normalized incomplete HFLPR.

**Definition** **8.** *For an IHFLPR $B={\left(b_{ij}\right)}_{n\times n}$, after utilizing δ(0≤δ≤1) to add some linguistic terms to $b_{ij}\in \Phi (i<j)$ and 1−δ to add linguistic terms to $b_{ji}\in \Phi (i<j)$, we can obtain a normalized IHFLPR, $\bar{B}={\left({\bar{b}}_{ij}\right)}_{n\times n}$, satisfying*

(10) $$L_{{\bar{b}}_{ij}}=\max\{L_{b_{ij}}|i,j=1,2,\cdots ,n,\;i\neq j,\;{\bar{b}}_{ij}\in \Phi \}$$

*where $L_{{\bar{b}}_{ij}}$ is the number of linguistic terms in ${\bar{b}}_{ij}$*.

**Example** **3.** *Let δ=1, a normalized IHFLPR is obtained for B given above*

$$\bar{B}=\left\{\begin{array}{cccc} \left\{s_{0}\right\} & x & \left\{s_{2},s_{3},s_{3}\right\} & x\\ x & \left\{s_{0}\right\} & x & \left\{s_{-4},s_{-3},s_{-2}\right\}\\ \left\{s_{-2},s_{-3},s_{-3}\right\} & x & \left\{s_{0}\right\} & \left\{s_{4},s_{4},s_{4}\right\}\\ x & \left\{s_{4},s_{3},s_{2}\right\} & \left\{s_{-4},s_{-4},s_{-4}\right\} & \left\{s_{0}\right\} \end{array}\right\}$$

**Definition** **9.** *For an IHFLPR $B={\left(b_{ij}\right)}_{n\times n}$, the elements $b_{ij}$ and $b_{kl}$ are adjacent if (i,j)∩(k,l)≠∅. For the missing element $b_{ij}$, if there exists adjacent complete elements $b_{ij_{1}},b_{j_{1}j_{2}},\cdots ,b_{j_{k}j}$, then $b_{ij}$ can be obtained indirectly*.

**Definition** **10.** *An IHFLPR $B={\left(b_{ij}\right)}_{n\times n}$ is acceptable if any missing elements of it can be obtained indirectly through the given elements; otherwise, it is an unacceptable IHFLPR*.

**Theorem** **3.** *A necessary condition for the acceptable IHFLPR is that, except for diagonal elements, there is at least one element in each column or each row with at least n−1 judgments*.

**Proof.**  Let $b_{kl}$ be a missing element in the IHFLPR $B={\left(b_{ij}\right)}_{n\times n}$. When B is acceptable, based on Definition 10, there exists a known complementary element sequence $b_{kl_{1}},b_{l_{1}l_{2}},\ldots ,b_{l_{t}l}$. Therefore, there is at least one given element $b_{kl_{1}}$ in the kth row, and $k\neq l_{1}$. Likewise, there exists at least one given element $b_{l_{t}l}$, and $l_{t}\neq l$. Due to the arbitrariness of k and l, the necessary condition can be confirmed. Thus, the upper (lower) triangle of incomplete HFLPR has at least n−1 judgments. ☐

**Definition** **11.** *Let $B={\left(b_{ij}\right)}_{n\times n}$ be an IHFLPR and its normalized IHFLPR with δ is $\bar{B}={\left({\bar{b}}_{ij}\right)}_{n\times n}$. Then B is called an additive consistent IHFLPR if the known elements of $\bar{B}$ satisfy*

(11) $${\bar{b}}_{ij_{1}}^{\sigma (s)}⊕{\bar{b}}_{j_{1}j_{2}}^{\sigma (s)}⊕\cdots +{\bar{b}}_{j_{t-1}j_{t}}^{\sigma (s)}⊕{\bar{b}}_{j_{t}j_{i}}^{\sigma (s)}=s_{0},\;i<j_{1}<j_{2}<\cdots <j_{t}$$

### 4.2. An Estimation Process for Acceptable IHFLPR with Only n−1 Judgments

After obtaining the normalized IHFLPR, the next work is to estimate the missing elements of it. Algorithm 1 is introduced to estimate the missing information of the IHFLPR with n−1 known judgments over all alternatives (one particular alternative is compared with all other alternatives only once).


**Algorithm 1. Estimating missing information of IHFLPR with n−1 known judgments**
  **Step 1.** There are a set of alternatives $X=\{x_{1,}x_{2},\cdots ,x_{n}\}$. Because of various reasons, an expert only compares one alternative with the other n−1 alternatives. The comparison information of the other n−1 alternatives is missing. Thus, n−1 judgments related to all alternatives can be obtained. An acceptable IHFLPR is constructed by these n−1 judgments. Go to Step 2.  **Step 2.** Obtain the normalized IHFLPR based on Definition 8. Go to Step 3.  **Step 3.** Estimate the missing elements of the normalized IHFLPR by Theorems 1 and 2. We can obtain a new additive consistent HFLPR ${\bar{B}}^{\prime }={\left({\bar{b}}_{ij}^{\prime }\right)}_{n\times n}$. If there are linguistic terms that are not in $\left\{s_{\alpha }|\alpha \in [-\tau ,\tau ]\right\}$, then the transformation function F(x)=τx/(τ+2a) can be used to preserve the additive consistency and reciprocity, resulting in a new consistent HFLPR ${\bar{B}}^{\prime \prime }={\left({\bar{b}}_{ij}^{\prime \prime }\right)}_{n\times n}$.**Step 4.** End.

**Example** **4.** *There are a set of five alternatives $x_{i}(i=1,2,\cdots ,5)$. One expert compares the second alternative with the other four alternatives and provides four judgments as follows: $b_{21}=\{s_{0},s_{1},s_{2}\}$, $b_{23}=\{s_{-2},s_{-3}\}$, $b_{24}=\{s_{1},s_{2},s_{3},s_{4}\}$ and $b_{25}=\{s_{-3},s_{-4}\}$. Algorithm 1 is used to estimate the missing elements of this IHFLPR*.

**Step 1.** Obtain the acceptable IHFLPR B through the four judgements provided by the expert:

$$B=\left\{\begin{array}{ccccc} \left\{s_{0}\right\} & \left\{s_{-2},s_{-1},s_{0}\right\} & x & x & x\\ \left\{s_{2},s_{1},s_{0}\right\} & \left\{s_{0}\right\} & \left\{s_{-3},s_{-2}\right\} & \left\{s_{1},s_{2},s_{3},s_{4}\right\} & \left\{s_{-4},s_{-3}\right\}\\ x & \left\{s_{3},s_{2}\right\} & \left\{s_{0}\right\} & x & x\\ x & \left\{s_{-1},s_{-2},s_{-3},s_{-4}\right\} & x & \left\{s_{0}\right\} & x\\ x & \left\{s_{4},s_{3}\right\} & x & x & \left\{s_{0}\right\} \end{array}\right\}$$

**Step 2.** Transform $B={\left(b_{ij}\right)}_{5\times 5}$ into a normalization IHFLPR based on Definition 8. Let δ=1. The normalized IHFLPR $\bar{B}={\left({\bar{b}}_{ij}\right)}_{5\times 5}$ is derived as

$$\bar{B}=\left\{\begin{array}{ccccc} \left\{s_{0}\right\} & \left\{s_{-2},s_{-1},s_{0},s_{0}\right\} & x & x & x\\ \left\{s_{2},s_{1},s_{0},s_{0}\right\} & \left\{s_{0}\right\} & \left\{s_{-3},s_{-2},s_{-2},s_{-2}\right\} & \left\{s_{1},s_{2},s_{3},s_{4}\right\} & \left\{s_{-4},s_{-3},s_{-3},s_{-3}\right\}\\ x & \left\{s_{3},s_{2},s_{2},s_{2}\right\} & \left\{s_{0}\right\} & x & x\\ x & \left\{s_{-1},s_{-2},s_{-3},s_{-4}\right\} & x & \left\{s_{0}\right\} & x\\ x & \left\{s_{4},s_{3},s_{3},s_{3}\right\} & x & x & \left\{s_{0}\right\} \end{array}\right\}$$

**Step 3.** Estimate the unknown elements of the normalized IHFLPR $\bar{B}={\left({\bar{b}}_{ij}\right)}_{5\times 5}$ based on Theorems 1 and 2. For example, to estimate ${\bar{b}}_{31}$ and ${\bar{b}}_{13}$, the procedure is presented as follows:

$$\begin{array}{r} I\left({\bar{b}}_{31}^{\sigma (1)}\right)=-I\left({\bar{b}}_{12}^{\sigma (1)}\right)-I\left({\bar{b}}_{23}^{\sigma (1)}\right)=5,\;I\left({\bar{b}}_{31}^{\sigma (2)}\right)=3,\;I\left({\bar{b}}_{31}^{\sigma (3)}\right)=2,\;I\left({\bar{b}}_{31}^{\sigma (4)}\right)=2,\\ I\left({\bar{b}}_{13}^{\sigma (1)}\right)=-5,\;I\left({\bar{b}}_{13}^{\sigma (2)}\right)=-3,\;I\left({\bar{b}}_{13}^{\sigma (3)}\right)=-2,\;I\left({\bar{b}}_{13}^{\sigma (4)}\right)=-2. \end{array}$$

Using the same process the rest unknown elements of $\bar{B}$ and the complete HFLPR can be obtained as

$${\bar{B}}^{\prime }=\left\{\begin{array}{ccccc} \left\{s_{0}\right\} & \left\{s_{-2},s_{-1},s_{0},s_{0}\right\} & \left\{s_{-5},s_{-3},s_{-2},s_{-2}\right\} & \left\{s_{-1},s_{1},s_{3},s_{4}\right\} & \left\{s_{-6},s_{-4},s_{-3},s_{-3}\right\}\\ \left\{s_{2},s_{1},s_{0},s_{0}\right\} & \left\{s_{0}\right\} & \left\{s_{-3},s_{-2},s_{-2},s_{-2}\right\} & \left\{s_{1},s_{2},s_{3},s_{4}\right\} & \left\{s_{-4},s_{-3},s_{-3},s_{-3}\right\}\\ \left\{s_{5},s_{3},s_{2},s_{2}\right\} & \left\{s_{3},s_{2},s_{2},s_{2}\right\} & \left\{s_{0}\right\} & \left\{s_{4},s_{4},s_{5},s_{6}\right\} & \left\{s_{-1},s_{-1},s_{-1},s_{-1}\right\}\\ \left\{s_{1},s_{-1},s_{-3},s_{-4}\right\} & \left\{s_{-1},s_{-2},s_{-3},s_{-4}\right\} & \left\{s_{-4},s_{-4},s_{-5},s_{-6}\right\} & \left\{s_{0}\right\} & \left\{s_{-5},s_{-5},s_{-6},s_{-7}\right\}\\ \left\{s_{6},s_{4},s_{3},s_{3}\right\} & \left\{s_{4},s_{3},s_{3},s_{3}\right\} & \left\{s_{1},s_{1},s_{1},s_{1}\right\} & \left\{s_{5},s_{5},s_{6},s_{7}\right\} & \left\{s_{0}\right\} \end{array}\right\}$$

It is observed that some linguistic terms are not in $\left\{s_{\alpha }|\alpha \in [-4,4]\right\}$. Thus, the transformation function F(x)=τx/(τ+a) is used to construct a new complete HFLPR and here a=7−4=3.

$${\bar{B}}^{\prime \prime }=\{\begin{array}{ccccc} \left\{s_{0}\right\} & \left\{s_{-1.14},s_{-0.57},s_{0},s_{0}\right\} & \left\{s_{-2.86},s_{-1.71},s_{-1.14},s_{-1.14}\right\} & \left\{s_{-0.57},s_{0.57},s_{1.71},s_{2.29}\right\} & \left\{s_{-3.43},s_{-2.29},s_{-1.71},s_{-1.71}\right\}\\ \left\{s_{1.14},s_{0.57},s_{0},s_{0}\right\} & \left\{s_{0}\right\} & \left\{s_{-1.71},s_{-1.14},s_{-1.14},s_{-1.14}\right\} & \left\{s_{0.57},s_{1.14},s_{1.71},s_{2.29}\right\} & \left\{s_{-2.29},s_{-1.71},s_{-1.71},s_{-1.71}\right\}\\ \left\{s_{2.86},s_{1.71},s_{1.71},s_{1.71}\right\} & \left\{s_{1.71},s_{1.14},s_{1.14},s_{1.14}\right\} & \left\{s_{0}\right\} & \left\{s_{2.29},s_{2.29},s_{2.86},s_{2.86}\right\} & \left\{s_{-0.57},s_{-0.57},s_{-0.57},s_{-0.57}\right\}\\ \left\{s_{0.57},s_{-0.57},s_{-1.71},s_{-2.29}\right\} & \left\{s_{-0.57},s_{-1.14},s_{-1.71},s_{-2.29}\right\} & \left\{s_{-2.29},s_{-2.29},s_{-2.29},s_{-3.43}\right\} & \left\{s_{0}\right\} & \left\{s_{-2.86},s_{-2.86},s_{-3.43},s_{-4}\right\}\\ \left\{s_{3.43},s_{2.29},s_{1.71},s_{1.71}\right\} & \left\{s_{2.29},s_{1.71},s_{1.71},s_{1.71}\right\} & \left\{s_{0.57},s_{0.57},s_{0.57},s_{0.57}\right\} & \left\{s_{2.86},s_{2.86},s_{3.43},s_{4}\right\} & \left\{s_{0}\right\} \end{array}\}$$

### 4.3. An Estimation Process for Acceptable IHFLPR with More Than n−1 Judgments

The process to estimate the missing elements of the IHFLPR with n−1 judgments has been discussed. In this subsection, we estimate the missing elements of the IHFLPR with more than n−1 known judgments. First, the IHFLPR is transformed into a normalized IHFLPR based on Definition 8. Then, a sequence of HFLEs ${\bar{b}}_{ij_{1}},{\bar{b}}_{ij_{2}},\cdots ,{\bar{b}}_{ij_{t}}(i<j_{1}<j_{2}<\cdots <j_{t})$ that includes only one HFLE ${\bar{b}}_{j_{k-1}j_{k}}$ is found. If $j_{k-1}<j_{k}$, then ${\bar{b}}_{j_{k-1}j_{k}}$ is in the middle of the sequence. In this case, based on Definition 11, the missing element ${\bar{b}}_{j_{k-1}j_{k}}$ can be estimated as follows:

(12) $$I\left({\bar{b}}^{\prime }{}_{j_{k-1}j_{k}}^{\sigma (l)}\right)=-\frac{1}{L}\sum_{i<j_{1}<j_{2}<\cdots <j_{t}}\left(I\left({\bar{b}}_{ij_{1}}^{\sigma (l)}\right)+I\left({\bar{b}}_{j_{1}j_{2}}^{\sigma (l)}\right)+\cdots +I\left({\bar{b}}_{j_{k-2}j_{k-1}}^{\sigma (l)}\right)+I\left({\bar{b}}_{j_{k}j_{k+1}}^{\sigma (l)}\right)+\cdots +I\left({\bar{b}}_{j_{t-1}j_{t}}^{\sigma (l)}\right)+I\left({\bar{b}}_{j_{t}i}^{\sigma (l)}\right)\right)$$

where ${\bar{b}}_{ij_{1}},\;{\bar{b}}_{j_{1}j_{2}},\cdots ,{\bar{b}}_{j_{k-2}j_{k-1}},\;{\bar{b}}_{j_{k}j_{k+1}},\cdots ,{\bar{b}}_{j_{t-1}j_{t}}^{\sigma (l)},\;{\bar{b}}_{j_{t}i}^{\sigma (l)}\in \Phi$ and L is the number of eligible sequences that include the missing element ${\bar{b}}_{j_{k-1}j_{k}}$.

If $j_{k-1}>j_{k}$, then ${\bar{b}}_{j_{k-1}j_{k}}$ is the last element in the sequence. In this case, ${\bar{b}}_{j_{k-1}j_{k}}$ is equal to ${\bar{b}}_{j_{t}i}^{\sigma (l)}$, and thus, the missing element can be estimated by the following equation:

(13) $$I\left({\bar{b}}^{\prime }{}_{j_{k-1}j_{k}}^{\sigma (l)}\right)=-\frac{1}{L}\sum_{i<j_{1}<j_{2}<\cdots <j_{t}}\left(I\left({\bar{b}}_{ij_{1}}^{\sigma (l)}\right)+I\left({\bar{b}}_{j_{1}j_{2}}^{\sigma (l)}\right)+\cdots +I\left({\bar{b}}_{j_{t-1}j_{t}}^{\sigma (l)}\right)+I\left({\bar{b}}_{j_{t}i}^{\sigma (l)}\right)\right)$$

Next, the above procedure is illustrated by an example:

**Example** **5.** *Assume that an IHFLPR with more than n−1 known judgments is given as:*

$$B=\left\{\begin{array}{ccccc} \left\{s_{0}\right\} & \left\{s_{2},s_{3},s_{4}\right\} & \left\{s_{-2},s_{-1}\right\} & \left\{s_{-1}\right\} & x\\ \left\{s_{-2},s_{-3},s_{-4}\right\} & \left\{s_{0}\right\} & \left\{s_{-3},s_{-2}\right\} & x & \left\{s_{-2},s_{-1}\right\}\\ \left\{s_{2},s_{1}\right\} & \left\{s_{3},s_{2}\right\} & \left\{s_{0}\right\} & x & \left\{s_{1}\right\}\\ \left\{s_{1}\right\} & x & x & \left\{s_{0}\right\} & \left\{s_{0},s_{1},s_{2},s_{3}\right\}\\ x & \left\{s_{2},s_{1}\right\} & \left\{s_{-1}\right\} & \left\{s_{0},s_{-1},s_{-2},s_{-3}\right\} & \left\{s_{0}\right\} \end{array}\right\}$$

First, Definition 8 is used to transform B into a normalized IHFLPR $\bar{B}$. Let δ=1. We have

$$\bar{B}=\left\{\begin{array}{ccccc} \left\{s_{0}\right\} & \left\{s_{2},s_{3},s_{4},s_{4}\right\} & \left\{s_{-2},s_{-1},s_{-1},s_{-1}\right\} & \left\{s_{-1},s_{-1},s_{-1},s_{-1}\right\} & x\\ \left\{s_{-2},s_{-3},s_{-4},s_{-4}\right\} & \left\{s_{0}\right\} & \left\{s_{-3},s_{-2},s_{-2},s_{-2}\right\} & x & \left\{s_{-2},s_{-1},s_{-1},s_{-1}\right\}\\ \left\{s_{2},s_{1},s_{1},s_{1}\right\} & \left\{s_{3},s_{2},s_{2},s_{2}\right\} & \left\{s_{0}\right\} & x & \left\{s_{1},s_{1},s_{1},s_{1}\right\}\\ \left\{s_{1},s_{1},s_{1},s_{1}\right\} & x & x & \left\{s_{0}\right\} & \left\{s_{0},s_{1},s_{2},s_{3}\right\}\\ x & \left\{s_{2},s_{1},s_{1},s_{1}\right\} & \left\{s_{-1},s_{-1},s_{-1},s_{-1}\right\} & \left\{s_{0},s_{-1},s_{-2},s_{-3}\right\} & \left\{s_{0}\right\} \end{array}\right\}$$

We first estimate the missing element ${\bar{b}}_{24}$. There is only one sequence that includes only one element ${\bar{b}}_{24}$: $\left\{{\bar{b}}_{12},{\bar{b}}_{24},{\bar{b}}_{41}\right\}$. ${\bar{b}}_{24}$ is in the middle of the sequence. Based on Equation (12), we have$I\left({\bar{b}}_{24}^{\sigma (1)}\right)=$$-I\left({\bar{b}}_{12}^{\sigma (1)}\right)--I\left({\bar{b}}_{41}^{\sigma (1)}\right)=-3$, $I\left({\bar{b}}_{24}^{\sigma (2)}\right)=-4$, $I\left({\bar{b}}_{24}^{\sigma (3)}\right)=-5$, $I\left({\bar{b}}_{24}^{\sigma (4)}\right)=-5$, $I\left({\bar{b}}_{42}^{\sigma (1)}\right)=3$, $I\left({\bar{b}}_{42}^{\sigma (2)}\right)=4$, $I\left({\bar{b}}_{42}^{\sigma (3)}\right)=5$, $I\left({\bar{b}}_{42}^{\sigma (4)}\right)=5$.

Next, we estimate the missing element ${\bar{b}}_{34}^{}$. There are two sequences that include ${\bar{b}}_{34}$: $\left\{{\bar{b}}_{12},{\bar{b}}_{23},{\bar{b}}_{34},{\bar{b}}_{41}\right\}$ and $\left\{{\bar{b}}_{13},{\bar{b}}_{34},{\bar{b}}_{41}\right\}$. ${\bar{b}}_{34}$ is in the middle of these two sequences. Based on Equation (12), we have $I\left({\bar{b}}_{34}^{\sigma (1)}\right)=\frac{1}{2}\left(\left(-I\left({\bar{b}}_{12}^{\sigma (1)}\right)-I\left({\bar{b}}_{23}^{\sigma (1)}\right)-I\left({\bar{b}}_{41}^{\sigma (1)}\right)\right)+\left(-I\left({\bar{b}}_{13}^{\sigma (1)}\right)-I\left({\bar{b}}_{41}^{\sigma (1)}\right)\right)\right)=0.5$, $I\left({\bar{b}}_{34}^{\sigma (2)}\right)=-1$, $I\left({\bar{b}}_{34}^{\sigma (3)}\right)=-0.5$, $I\left({\bar{b}}_{34}^{\sigma (4)}\right)=-0.5$, $I\left({\bar{b}}_{43}^{\sigma (1)}\right)=-0.5$, $I\left({\bar{b}}_{43}^{\sigma (2)}\right)=1$, $I\left({\bar{b}}_{43}^{\sigma (3)}\right)=0.5$, $I\left({\bar{b}}_{43}^{\sigma (4)}\right)=0.5$.

For the missing element ${\bar{b}}_{51}$, there is only one sequence that include ${\bar{b}}_{51}$, i.e., $\left\{{\bar{b}}_{12}^{},{\bar{b}}_{25}^{},{\bar{b}}_{51}^{}\right\}$. ${\bar{b}}_{51}$ is the last element of this sequence. Based on Equation (13), we have $I\left({\bar{b}}_{51}^{\sigma (1)}\right)=-I\left({\bar{b}}_{12}^{\sigma (1)}\right)--I\left({\bar{b}}_{25}^{\sigma (1)}\right)=0$, $I\left({\bar{b}}_{51}^{\sigma (2)}\right)=-2$, $I\left({\bar{b}}_{51}^{\sigma (3)}\right)=-3$, $I\left({\bar{b}}_{51}^{\sigma (4)}\right)=-3$, $I\left({\bar{b}}_{51}^{\sigma (1)}\right)=0$, $I\left({\bar{b}}_{51}^{\sigma (2)}\right)=2$, $I\left({\bar{b}}_{51}^{\sigma (3)}\right)=3$, $I\left({\bar{b}}_{51}^{\sigma (4)}\right)=3$. Then, we have

$${\bar{B}}^{\prime }=\left\{\begin{array}{ccccc} \left\{s_{0}\right\} & \left\{s_{2},s_{3},s_{4},s_{4}\right\} & \left\{s_{-2},s_{-1},s_{-1},s_{-1}\right\} & \left\{s_{-1},s_{-1},s_{-1},s_{-1}\right\} & \left\{s_{0},s_{2},s_{3},s_{3}\right\}\\ \left\{s_{-2},s_{-3},s_{-4},s_{-4}\right\} & \left\{s_{0}\right\} & \left\{s_{-3},s_{-2},s_{-2},s_{-2}\right\} & \left\{s_{-3},s_{-4},s_{-5},s_{-5}\right\} & \left\{s_{-2},s_{-1},s_{-1},s_{-1}\right\}\\ \left\{s_{2},s_{1},s_{1},s_{1}\right\} & \left\{s_{3},s_{2},s_{2},s_{2}\right\} & \left\{s_{0}\right\} & \left\{s_{0.5},s_{-1},s_{-0.5},s_{-0.5}\right\} & \left\{s_{1},s_{1},s_{1},s_{1}\right\}\\ \left\{s_{1},s_{1},s_{1},s_{1}\right\} & \left\{s_{3},s_{4},s_{5},s_{5}\right\} & \left\{s_{-0.5},s_{1},s_{0.5},s_{0.5}\right\} & \left\{s_{0}\right\} & \left\{s_{0},s_{1},s_{2},s_{3}\right\}\\ \left\{s_{0},s_{-2},s_{-3},s_{-3}\right\} & \left\{s_{2},s_{1},s_{1},s_{1}\right\} & \left\{s_{-1},s_{-1},s_{-1},s_{-1}\right\} & \left\{s_{0},s_{-1},s_{-2},s_{-3}\right\} & \left\{s_{0}\right\} \end{array}\right\},$$

As some linguistic values in ${\bar{B}}^{\prime }$ are not in $\left\{s_{\alpha }|\alpha \in [-4,4]\right\}$, the transformation function F(x)=τx/(τ+2a) can be used to construct a new HFLPR ${\bar{B}}^{\prime \prime }$ with a=5−4=1.

$${\bar{B}}^{\prime \prime }=\left\{\begin{array}{ccccc} \left\{s_{0}\right\} & \left\{s_{1.6},s_{2.4},s_{3.2},s_{3.2}\right\} & \left\{s_{-1.6},s_{-0.8},s_{-0.8},s_{-0.8}\right\} & \left\{s_{-0.8},s_{-0.8},s_{-0.8},s_{-0.8}\right\} & \left\{s_{0},s_{1.6},s_{2.4},s_{2.4}\right\}\\ \left\{s_{-1.6},s_{-2.4},s_{-3.2},s_{-3.2}\right\} & \left\{s_{0}\right\} & \left\{s_{-2.4},s_{-1.6},s_{-1.6},s_{-1.6}\right\} & \left\{s_{-2.4},s_{-3.2},s_{-4},s_{-4}\right\} & \left\{s_{-1.6},s_{-0.8},s_{-0.8},s_{-0.8}\right\}\\ \left\{s_{1.6},s_{0.8},s_{0.8},s_{0.8}\right\} & \left\{s_{2.4},s_{1.6},s_{1.6},s_{1.6}\right\} & \left\{s_{0}\right\} & \left\{s_{0.4},s_{-0.8},s_{-0.4},s_{-0.4}\right\} & \left\{s_{0.8},s_{0.8},s_{0.8},s_{0.8}\right\}\\ \left\{s_{0.8},s_{0.8},s_{0.8},s_{0.8}\right\} & \left\{s_{2.4},s_{3.2},s_{4},s_{4}\right\} & \left\{s_{-0.4},s_{0.8},s_{0.4},s_{0.4}\right\} & \left\{s_{0}\right\} & \left\{s_{0},s_{0.8},s_{1.6},s_{2.4}\right\}\\ \left\{s_{0},s_{-1.6},s_{-2.4},s_{-2.4}\right\} & \left\{s_{1.6},s_{0.8},s_{0.8},s_{0.8}\right\} & \left\{s_{-0.8},s_{-0.8},s_{-0.8},s_{-0.8}\right\} & \left\{s_{0},s_{-0.8},s_{-1.6},s_{-2.4}\right\} & \left\{s_{0}\right\} \end{array}\right\}$$

### 4.4. A Strategy to Deal with Ignorance Situations

The situation in which each alternative is compared at least once has been discussed. However, there may also be the case in which an expert does not give any preference information of the alternatives as the expert does not have a precise understanding of the problem. The expert cannot determine which options are superior to others. This case is called a total ignorance situation and it is necessary for us to go further and manage such a situation. Alonso et al. [22] proposed five strategies to estimate the missing elements in a total ignorance situation. Based on this, in this subsection, a strategy which is called social strategy is introduced to address such a situation.

Let $X=\{x_{1},x_{2},\cdots ,x_{n}\}$ be a set of alternatives and $E=\{E_{1},E_{2},\cdots ,E_{m}\}$ be m experts. We assume that $B_{h}$ is an IHFLPR provided by the expert $E_{h}$ with unknown evaluations for the alternative $x_{i}$, that is, $b_{ij}^{\left(h\right)}$ and $b_{ji}^{\left(h\right)}$ are unknown for all j=1,2,⋯,n. The first task we should do, is to compute the distance between $B_{h}$ and the other HFLPRs. Zhu and Xu [14] defined the distance between two HFLEs as

(14) $$d(b_{\alpha },b_{\beta })=\frac{S_{\varepsilon }\left(I\left(b_{\alpha }\right)-I\left(b_{\beta }\right)\right)}{L_{b}(2\tau +1)}$$

where $S_{\varepsilon }$ is a function denotes the sum of all linguistic terms in a set and $L_{b}=L_{b_{\alpha }}=L_{b_{\beta }}$ is the number of linguistic terms for the HFLEs. However, there is a flaw in Equation (14). For example, if two experts’ opinions are completely opposed, which can be represented in HFLE as $b_{1}=\{s_{-4}\}$ and $b_{2}=\{s_{4}\}$, then the distance between them should be 1. While the result calculated by Equation (14) is $\frac{8}{9}\neq 1$, which is not logical. Thus, below a novel distance between two HFLEs is defined.

**Definition** **12.** *Let S be a LTS, $b_{\alpha }$ and $b_{\beta }$$\left(L_{b_{\alpha }}=L_{b_{\beta }}\right)$ be two HFLEs on S. The distance between $b_{\alpha }$ and $b_{\beta }$ is defined as*

(15) $$d(b_{\alpha },b_{\beta })=\frac{S_{\varepsilon }\left(I\left(b_{\alpha }\right)-I\left(b_{\beta }\right)\right)}{2\tau L_{b}}$$

*where $S_{\varepsilon }$ is a function that denotes the sum of all values in a set and $L_{b}=L_{b_{\alpha }}=L_{b_{\beta }}$*.

Based on Definition 12, a distance measure between two HFLPRs in Definition 13 is introduced.

**Definition** **13.** *Let $B_{\eta }$ and $B_{\mu }$ be two HFLPRs, and their corresponding normalized HFLPRs are ${\bar{B}}_{\eta }={\left({\bar{b}}_{ij}\right)}_{n\times n}^{\eta }$ and ${\bar{B}}_{\mu }={\left({\bar{b}}_{ij}\right)}_{n\times n}^{\mu }$, respectively. The distance between $B_{\eta }$ and $B_{\mu }$ is*

(16) $$d\left(B_{\eta },B_{\mu }\right)=\sqrt{\frac{2}{n(n-1)}\sum_{i=1,i<j}^{n}{\left(d\left({\left({\bar{b}}_{ij}\right)}_{\eta },{\left({\bar{b}}_{ij}\right)}_{\mu }\right)\right)}^{2}}$$

**Theorem** **4.** *For two HFLPRs $B_{\eta }$ and $B_{\mu }$, their distance $d\left(B_{\eta },B_{\mu }\right)$ satisfies*

(1) $0\leq d\left(B_{\eta },B_{\mu }\right)\leq 1$;
(2) $d\left(B_{\eta },B_{\mu }\right)=0$*if and only if*$B_{\eta }=B_{\mu }$;
(3) $d\left(B_{\eta },B_{\mu }\right)=d\left(B_{\mu },B_{\beta }\right)$.

If there are unknown elements in the HFLPRs, the distance between an incomplete HFLPR and a complete HFLPR is defined.

**Definition** **14.** *For an IHFLPR $B_{\alpha }$ and a complete HFLPR $B_{\beta }$, and their corresponding normalized IHFLPR ${\bar{B}}_{\alpha }={\left({\bar{b}}_{ij}\right)}_{n\times n}^{\alpha }$ and HFLPR ${\bar{B}}_{\beta }={\left({\bar{b}}_{ij}\right)}_{n\times n}^{\beta }$, the distance between $B_{\alpha }$ and $B_{\beta }$ is*

(17) $$d\left(B_{\alpha },B_{\beta }\right)=\sqrt{\frac{2}{L_{\Phi }}\sum_{i=1,i<j}^{n}{\left(d\left({\left({\bar{b}}_{ij}\right)}_{\alpha },{\left({\bar{b}}_{ij}\right)}_{\beta }\right)\right)}^{2}}$$

*where $L_{\Phi }$ is the number of known elements in $B_{\alpha }$ excluding diagonal elements*.

Given a value ζ(0≤ζ≤1), Equation (17) is used to find the experts with a distance less than or equal to ζ from the expert $E_{h}$. The judgments of these experts are close to the expert $E_{h}$ and thus their HFLPRs can be used to estimate the unknown judgments of the alternative $x_{i}$ in $B_{h}$.

**Example** **6.** *Let $E_{t}(t=1,2,3,4)$ be four experts who are invited to solve a GDM problem over four alternatives $x_{1}$, $x_{2}$, $x_{3}$, and $x_{4}$. The expert $E_{1}$ provides his/her individual IHFLPR $B_{1}$ but does not provide any information about the alternative $x_{2}$:*

$$B_{1}=\left\{\begin{array}{cccc} \left\{s_{0}\right\} & x & \left\{s_{1},s_{2}\right\} & \left\{s_{2},s_{3}\right\}\\ x & \left\{s_{0}\right\} & x & x\\ \left\{s_{1},s_{2}\right\} & x & \left\{s_{0}\right\} & \left\{s_{2}\right\}\\ \left\{s_{-2},s_{-3}\right\} & x & \left\{s_{-2}\right\} & \left\{s_{0}\right\} \end{array}\right\}$$

The experts $E_{2}$, $E_{3}$ and $E_{4}$ provide the following HFLPRs:

$$B_{2}=\left\{\begin{array}{cccc} \left\{s_{0}\right\} & \left\{s_{1},s_{2}\right\} & \left\{s_{-2},s_{-1}\right\} & \left\{s_{3}\right\}\\ \left\{s_{-1},s_{-2}\right\} & \left\{s_{0}\right\} & \left\{s_{-2}\right\} & \left\{s_{0},s_{1}\right\}\\ \left\{s_{2},s_{1}\right\} & \left\{s_{2}\right\} & \left\{s_{0}\right\} & \left\{s_{3},s_{4}\right\}\\ \left\{s_{-3}\right\} & \left\{s_{0},s_{-1}\right\} & \left\{s_{-3},s_{-4}\right\} & \left\{s_{0}\right\} \end{array}\right\},$$

$$B_{3}=\left\{\begin{array}{cccc} \left\{s_{0}\right\} & \left\{s_{-2},s_{-1}\right\} & \left\{s_{-4}\right\} & \left\{s_{-1}\right\}\\ \left\{s_{2},s_{1}\right\} & \left\{s_{0}\right\} & \left\{s_{-2},s_{-1}\right\} & \left\{s_{1}\right\}\\ \left\{s_{4}\right\} & \left\{s_{2},s_{1}\right\} & \left\{s_{0}\right\} & \left\{s_{2},s_{3}\right\}\\ \left\{s_{1}\right\} & \left\{s_{-1}\right\} & \left\{s_{-2},s_{-3}\right\} & \left\{s_{0}\right\} \end{array}\right\},$$

$$B_{4}=\left\{\begin{array}{cccc} \left\{s_{0}\right\} & \left\{s_{0},s_{1}\right\} & \left\{s_{2}\right\} & \left\{s_{3},s_{4}\right\}\\ \left\{s_{0},s_{-1}\right\} & \left\{s_{0}\right\} & \left\{s_{-2},s_{-1}\right\} & \left\{s_{1}\right\}\\ \left\{s_{-2}\right\} & \left\{s_{2},s_{1}\right\} & \left\{s_{0}\right\} & \left\{s_{2},s_{3}\right\}\\ \left\{s_{-3},s_{-4}\right\} & \left\{s_{-1}\right\} & \left\{s_{-2},s_{-3}\right\} & \left\{s_{0}\right\} \end{array}\right\}$$

Then, these four HFLPRs are transformed into their corresponding normalized HFLPRs:

$${\bar{B}}_{1}=\left\{\begin{array}{cccc} \left\{s_{0}\right\} & x\{s_{1},s_{2}\} & \left\{s_{2},s_{3}\right\}\\ x & \left\{s_{0}\right\} & x & x\\ \left\{s_{1},s_{2}\right\} & x & \left\{s_{0}\right\} & \left\{s_{2},s_{2}\right\}\\ \left\{s_{-2},s_{-3}\right\} & x & \left\{s_{-2},s_{-2}\right\} & \left\{s_{0}\right\} \end{array}\right\},$$

$${\bar{B}}_{2}=\left\{\begin{array}{cccc} \left\{s_{0}\right\} & \left\{s_{1},s_{2}\right\} & \left\{s_{-2},s_{-1}\right\} & \left\{s_{3},s_{3}\right\}\\ \left\{s_{-1},s_{-2}\right\} & \left\{s_{0}\right\} & \left\{s_{-2},s_{-2}\right\} & \left\{s_{0},s_{1}\right\}\\ \left\{s_{2},s_{1}\right\} & \left\{s_{2},s_{2}\right\} & \left\{s_{0}\right\} & \left\{s_{3},s_{4}\right\}\\ \left\{s_{-3},s_{-3}\right\} & \left\{s_{0},s_{-1}\right\} & \left\{s_{-3},s_{-4}\right\} & \left\{s_{0}\right\} \end{array}\right\}$$

$${\bar{B}}_{3}=\left\{\begin{array}{cccc} \left\{s_{0}\right\} & \left\{s_{-2},s_{-1}\right\} & \left\{s_{-4},s_{-4}\right\} & \left\{s_{-1},s_{-1}\right\}\\ \left\{s_{2},s_{1}\right\} & \left\{s_{0}\right\} & \left\{s_{-2},s_{-1}\right\} & \left\{s_{1},s_{1}\right\}\\ \left\{s_{4},s_{4}\right\} & \left\{s_{2},s_{1}\right\} & \left\{s_{0}\right\} & \left\{s_{2},s_{3}\right\}\\ \left\{s_{1},s_{1}\right\} & \left\{s_{-1},s_{-1}\right\} & \left\{s_{-2},s_{-3}\right\} & \left\{s_{0}\right\} \end{array}\right\},$$

$${\bar{B}}_{4}=\left\{\begin{array}{cccc} \left\{s_{0}\right\} & \left\{s_{0},s_{1}\right\} & \left\{s_{2},s_{2}\right\} & \left\{s_{3},s_{4}\right\}\\ \left\{s_{0},s_{-1}\right\} & \left\{s_{0}\right\} & \left\{s_{-2},s_{-1}\right\} & \left\{s_{1},s_{1}\right\}\\ \left\{s_{-2},s_{-2}\right\} & \left\{s_{2},s_{1}\right\} & \left\{s_{0}\right\} & \left\{s_{2},s_{3}\right\}\\ \left\{s_{-3},s_{-4}\right\} & \left\{s_{-1},s_{-1}\right\} & \left\{s_{-2},s_{-3}\right\} & \left\{s_{0}\right\} \end{array}\right\}$$

Based on Equation (17), we calculate $d\left(B_{1},B_{2}\right)=0.2447$, $d\left(B_{1},B_{3}\right)=0.4719$, $d\left(B_{1},B_{4}\right)=0.0884$. Given ζ=0.25, the unknown elements of ${\bar{B}}_{1}$ can be calculated as the arithmetic means of the corresponding values in ${\bar{B}}_{2}$ and ${\bar{B}}_{4}$, and the value in ${\bar{B}}_{3}$ is not considered since the distance $d\left(B_{1},B_{3}\right)>\zeta$.

$$\left\{\begin{array}{cccc} \left\{s_{0}\right\} & x & \left\{s_{1},s_{2}\right\} & \left\{s_{2},s_{3}\right\}\\ x & \left\{s_{0}\right\} & x & x\\ \left\{s_{1},s_{2}\right\} & x & \left\{s_{0}\right\} & \left\{s_{2},s_{2}\right\}\\ \left\{s_{-2},s_{-3}\right\} & x & \left\{s_{-2},s_{2}\right\} & \left\{s_{0}\right\} \end{array}\right\}\to \left\{\begin{array}{cccc} \left\{s_{0}\right\} & \left\{s_{0.5},s_{1.5}\right\} & \left\{s_{1},s_{2}\right\} & \left\{s_{2},s_{3}\right\}\\ \left\{s_{-0.5},s_{-1.5}\right\} & \left\{s_{0}\right\} & \left\{s_{-2},s_{-1.5}\right\} & \left\{s_{0.5},s_{1}\right\}\\ \left\{s_{1},s_{2}\right\} & \left\{s_{2},s_{1.5}\right\} & \left\{s_{0}\right\} & \left\{s_{2},s_{2}\right\}\\ \left\{s_{-2},s_{-3}\right\} & \left\{s_{-0.5},s_{-1}\right\} & \left\{s_{-2},s_{-2}\right\} & \left\{s_{0}\right\} \end{array}\right\}$$

## 5. A GDM Model with Incomplete HFLPRs

In this section, an algorithm for GDM with IHFLPRs is developed. To do so, a way to determine the weights of experts is defined firstly. A selection process to rank alternatives and select the optimal one is also proposed.

### 5.1. Determining the Weights of Experts

When determining the experts’ weights, the completeness of the IHFLPRs should be taken into consideration. An expert should be endowed a larger weight if he can provide more known information. Zhao et al. [23] introduced the concept of the quality of a linguistic preference relation and proposed the experience and quality hybrid weight vector. To evaluate the experts’ weights in the environment of IHFLPRs, the concept of the quality of an IHFLPR is introduced.

**Definition** **15.** *Let $B={\left(b_{ij}\right)}_{n\times n}$ be an IHFLPR. The quality of B is defined as*

(18) $$Q_{B}=\frac{2L_{\Phi }}{n(n-1)}$$

*where $L_{\Phi }$ is the number of known elements in the upper triangle of B*.

For instance, in Example 6, the quality of $B_{1}$ is $Q_{B_{1}}$ = $\frac{2\times 3}{4\times 3}=\frac{1}{2}$. Obviously, $Q_{B}\in [0,1]$. If all elements are known, then $Q_{B}=1$. If all elements are unknown, then $Q_{B}=0$.

Based on the quality of the IHFLPR, the weight $u_{t}$ of the expert $E_{t}$ regarding the qualities of IHFLPRs associated to the group can be determined as

(19) $$u_{t}=Q_{t}/\sum_{t=1}^{m}Q_{t},\;t=1,2,\cdots ,m$$

**Definition** **16.** *For a GDM problem with m experts, their subjective weight vector is $\lambda ={\left(\lambda_{1},\lambda_{2},\ldots ,\lambda_{m}\right)}^{T}$, $\sum_{t=1}^{m}\lambda_{t}=1$. Their weight vector regarding to the quality of IHFLPR is $u={\left(u_{1},u_{2},\ldots ,u_{m}\right)}^{T}$, $\sum_{t=1}^{m}u_{t}=1$. The comprehensive weight $\omega_{t}$ of the expert $E_{t}$ is defined as*

(20) $$\omega_{t}=\lambda_{t}u_{t}/\sum_{t=1}^{m}\lambda_{t}u_{t}$$

### 5.2. The Selection Process

After obtaining the complete HFLPRs and the weights of experts, we shall try to select the best alternative. There are two main phases in the selection process. The first one is the aggregation phase and the second one is the exploitation phase. The collective HFLPR from all the individual HFLPRs is derived and the global preferences over the alternatives is obtained in the aggregation phase. In the exploitation phase, a global ranking of the alternatives is determined.

#### 5.2.1. The Aggregation Phase

Once the missing elements of each IHFLPR are estimated, a set of individual complete HFLPRs can be derived. Then a collective HFLPR $B^{c}={\left(b_{ij}^{\left(c\right)}\right)}_{n\times n}$ can be obtained by the linguistic weighted averaging operator [5]:

(21) $$b_{ij}^{\sigma (l)(c)}=\omega_{1}b_{ij}^{\sigma (l)(1)}⊕\omega_{2}b_{ij}^{\sigma (l)(2)}⊕\cdots ⊕\omega_{m}b_{ij}^{\sigma (l)(m)},\;\mathrm{for}\;\mathrm{all}\;i,j=1,2,\ldots ,n$$

**Theorem** **5.** *If all the individual HFLPRs $B^{t}={\left(b_{ij}^{\left(t\right)}\right)}_{n\times n}$ (t=1,2,⋯,m) are additive consistent, then the collective HFLPR $B^{c}={\left(b_{ij}^{\left(c\right)}\right)}_{n\times n}$ is additive consistent*.

**Proof.**  Assume that $B^{t}={\left(b_{ij}^{\left(t\right)}\right)}_{n\times n}$ (t=1,2,…,m) are m additive consistent HFLPRs. For any i,j,k=1,2,…,n, i≠j≠k, we have

$$\begin{array}{l} b_{ij}^{\sigma (c)(2)}=\omega_{1}\left(b_{ik}^{\sigma (l)(1)}⊕b_{kj}^{\sigma (l)(1)}\right)⊕\omega_{2}\left(b_{ik}^{\sigma (l)(2)}⊕b_{kj}^{\sigma (l)(2)}\right)⊕\cdots ⊕\omega_{m}\left(b_{ik}^{\sigma (l)(m)}⊕b_{kj}^{\sigma (l)(m)}\right)\\ =\left(\omega_{1}b_{ik}^{\sigma (l)(1)}⊕\omega_{2}b_{ik}^{\sigma (l)(2)}⊕\cdots ⊕\omega_{m}b_{ik}^{\sigma (l)(m)}\right)⊕\left(\omega_{1}b_{kj}^{\sigma (l)(1)}⊕\omega_{2}b_{kj}^{\sigma (l)(2)}⊕\cdots ⊕\omega_{m}b_{kj}^{\sigma (l)(m)}\right) \end{array}$$

which denotes that $B^{c}={\left(b_{ij}^{\left(c\right)}\right)}_{n\times n}$ is additive consistent. ☐

#### 5.2.2. The Exploitation Phase

After fusing all the individual HFLPRs into a collective HFLPR, the preferences in the collective HFLPR regarding each alternative can be aggregated. Then, the ranking of the alternatives is derived by using the HFLWA operator [5].

### 5.3. An Algorithm for GDM with IHFLPRs

GDM takes an important role in many fields [24,25,26]. Owing to the increasing randomness and complexity of the decision-making environment, more and more organizations invite groups of experts to make decision instead of a single expert. The GDM problem with HFLPRs can be described as follows: Let $X=\{x_{1},x_{2},\ldots ,x_{n}\}$ be a set of alternatives, $E=\{E_{1},E_{2},\ldots ,E_{m}\}$ be a set of m experts. The subject weight vector of experts is $\lambda ={\left(\lambda_{1},\lambda_{2},\ldots ,\lambda_{m}\right)}^{T}$, where $\lambda_{t}>0$, t=1,2,…,m, and $\sum_{t=1}^{m}\lambda_{t}=1$. The subject weight vector can be determined by the experts’ experience. One expert compares each pair of alternatives and provides his/her preference relation over these alternatives. Due to the lack of knowledge or the heavy workload, some information is missing. Then, an IHFLPR $B={\left(b_{ij}\right)}_{n\times n}$ is constructed. Algorithm 2 is developed to address this problem.


**Algorithm 2. GDM with IHFLPRs**
  **Step 1.** Determine the alternatives $X=\{x_{1},x_{2},\ldots ,x_{n}\}$, the experts $E=\{E_{1},E_{2},\ldots ,E_{m}\}$, the subjective weight vector of the experts $\lambda ={\left(\lambda_{1},\lambda_{2},\ldots ,\lambda_{m}\right)}^{T}$, and the original IHFLPR $B^{t}={\left(b_{ij}^{\left(t\right)}\right)}_{n\times n}$ regarding each expert.  **Step 2.** Transform each IHFLPR $B^{t}={\left(b_{ij}^{\left(t\right)}\right)}_{n\times n}$ into the corresponding normalized IHFLPR ${\bar{B}}^{t}={\left({\bar{b}}_{ij}^{\left(t\right)}\right)}_{n\times n}$ with δ based on Definition 8. Then, these normalized IHFLPRs are classified into three categories: (1) the IHFLPRs with only n−1 judgments; (2) the IHFLPRs with more than n−1 known judgments; and (3) the IHFLPRs including an alternative $x_{i}$ that has no information.  **Step 3.** Estimate the missing information of the normalized IHFLPRs ${\bar{B}}^{t}={\left({\bar{b}}_{ij}^{\left(t\right)}\right)}_{n\times n}$ and obtain the complete HFLPRs ${\bar{B}}^{\prime }{}^{t}={\left({\bar{b}}^{\prime }{}_{ij}^{\left(t\right)}\right)}_{n\times n}$. If there are values that are not in $S=\{s_{\alpha }|\alpha \in [-\tau ,\tau ]\}$, the conversion function F(x)=τx/(τ+2a) is applied to convert all the linguistic terms in the complete HFLPRs to new HFLPRs.  **Step 4.** Determine the comprehensive weight vector of the experts by Equation (20).  **Step 5.** Use the linguistic weighted averaging operator shown as Equation (21) to fuse all the individual HFLPRs ${\bar{B}}^{\prime }{}^{t}={\left({\bar{b}}^{\prime }{}_{ij}^{\left(t\right)}\right)}_{n\times n}$ into a collective HFLPR ${\bar{B}}^{\prime }{}^{c}={\left({\bar{b}}^{\prime }{}_{ij}^{\left(c\right)}\right)}_{n\times n}$.  **Step 6.** Use the HFLWA operator shown as Equation (2) to fuse all the preference degrees ${\bar{b}}^{\prime }{}_{ij}^{\left(c\right)}$ in the ith line of ${\bar{B}}^{\prime c}$ and obtain the overall value of the ith alternative over all of other alternatives.  **Step 7.** Rank all the alternatives and choose the best one. Then, we end the algorithm.

## 6. Case Study and Comparative Analyses

In this section, a practical example in terms of flood disaster risk evaluation is used to illustrate our algorithm. A comprehensive comparative analysis is presented to verify the advantage of our GDM model.

### 6.1. Case Study about Flood Disaster Risk Evaluation

In recent years, with the change of the global environment and social and economic development, the risks of various natural disasters are increasing. All kinds of natural disasters cause huge losses and continue to threaten millions of people all over the world [27]. The United Nations International Strategy for Disaster Reduction reported that people should establish social systems that coexist with risks [28]. Against this background, natural disaster risk reduction management is the most effective and active way for disaster prevention. Disaster risk assessment is an important means of risk reduction management. This section selects flood disaster risk evaluation as the case study.

Up to now, some works related to the flood-risk evaluation have been carried out in China. Lai et al. [29] proposed an assessment model based on fuzzy comprehensive evaluation to evaluate flood disaster risk in the Dongjiang River Basin. Liu et al. [30] constructed a hydrological ensemble flood forecasting system to evaluate flood disaster risk in the Lanjiang Basin. Qi et al. [31] established a regional evaluation model of flood disasters based on the superposition theory.

The existing flood evaluation system is based on specific tools. Few studies focus on expert evaluation systems. In this paper, an expert evaluation model is established to evaluate flood disaster risk. Risk assessment in different regions is the basic content of our system. Experts’ knowledge and experience play a key role in the system. However, owing to the lack of comprehensive information, the experts may be unable to provide complete judgments when they assess pairwise alternatives. The unusually complex geographical and climatic conditions in China lead to the complexity of natural disaster systems and the spatial incompleteness of the data. Thus, the method proposed in this paper is suitable for disaster risk assessment. What is more, many disasters including floods need real-time assessment. The lag of assessment may lead to disastrous consequences. The flexibility of expert assessment can solve the problem of lag.

Suppose that there are five potentially flooded regions$X=\{x_{1},x_{2},x_{3},x_{4},x_{5}\}$. Four experts $E=\{E_{1},E_{2},E_{3},E_{4}\}$ (whose subject weight vector is $\lambda ={\left(0.25,0.25,0.25,0.25\right)}^{T}$) are invited to assess the flood disaster risks to make a purposeful precaution. The subject weight vector is determined by some prior information. It is a comprehensive quantity representation that includes experts’ knowledge, experiences, abilities, and expectation. Let ζ=0.20. Because of the lack of knowledge, some information is missing when the experts compare alternatives. These four experts provide their IHFLPRs as follows:

$$B_{1}=\left\{\begin{array}{ccccc} \left\{s_{0}\right\} & x & \left\{s_{-1}\right\} & x & x\\ x & \left\{s_{0}\right\} & \left\{s_{1},s_{2}\right\} & x & x\\ \left\{s_{1}\right\} & \left\{s_{-1},s_{-2}\right\} & \left\{s_{0}\right\} & \left\{s_{3}\right\} & \left\{s_{0},s_{1},s_{2}\right\}\\ x & x & \left\{s_{-3}\right\} & \left\{s_{0}\right\} & x\\ x & x & \left\{s_{0},s_{-1},s_{-2}\right\} & x & \left\{s_{0}\right\} \end{array}\right\}$$

$$B_{2}=\left\{\begin{array}{ccccc} \left\{s_{0}\right\} & \left\{s_{-1}\right\} & \left\{s_{0},s_{1}\right\} & \left\{s_{1},s_{2},s_{3}\right\} & \left\{s_{2},s_{3}\right\}\\ \left\{s_{1}\right\} & \left\{s_{0}\right\} & x & x & x\\ \left\{s_{0},s_{-1}\right\} & x & \left\{s_{0}\right\} & x & x\\ \left\{s_{-1},s_{-2},s_{-3}\right\} & x & x & \left\{s_{0}\right\} & x\\ \left\{s_{-2},s_{-3}\right\} & x & x & x & \left\{s_{0}\right\} \end{array}\right\}\;B_{3}=\left\{\begin{array}{ccccc} \left\{s_{0}\right\} & \left\{s_{1},s_{2},s_{3}\right\} & \left\{s_{3},s_{4}\right\} & \left\{s_{1},s_{2}\right\} & x\\ \left\{s_{-1},s_{-2},s_{-3}\right\} & \left\{s_{0}\right\} & \left\{s_{1}\right\} & x & \left\{s_{-2},s_{-1}\right\}\\ \left\{s_{-3},s_{-4}\right\} & \left\{s_{-1}\right\} & \left\{s_{0}\right\} & x & \left\{s_{-2}\right\}\\ \left\{s_{-1},s_{-2}\right\} & x & x & \left\{s_{0}\right\} & \left\{s_{-1},s_{0}\right\}\\ x & \left\{s_{2},s_{1}\right\} & \left\{s_{2}\right\} & \left\{s_{1},s_{0}\right\} & \left\{s_{0}\right\} \end{array}\right\}\;B_{4}=\left\{\begin{array}{ccccc} \left\{s_{0}\right\} & \left\{s_{-2}\right\} & \left\{s_{-1}\right\} & x & \left\{s_{0},s_{1},s_{2}\right\}\\ \left\{s_{2}\right\} & \left\{s_{0}\right\} & \left\{s_{0},s_{1}\right\} & x & \left\{s_{3},s_{4}\right\}\\ \left\{s_{1}\right\} & \left\{s_{0},s_{-1}\right\} & \left\{s_{0}\right\} & x & \left\{s_{3}\right\}\\ x & x & x & \left\{s_{0}\right\} & x\\ \left\{s_{0},s_{-1},s_{-2}\right\} & \left\{s_{-3},s_{-4}\right\} & \left\{s_{-3}\right\} & x & \left\{s_{0}\right\} \end{array}\right\}$$

Now, Algorithm 2 is used to select the area with the highest risk of flood. As Step 1 has been given above, the calculation starts from Step 2.

**Step 2.** Transform the IHFLPRs into their corresponding normalized IHFLPRs based on Definition 8. We assume δ=1 and obtain

$${\bar{B}}_{1}=\left\{\begin{array}{ccccc} \left\{s_{0}\right\} & x & \left\{s_{-1},s_{-1},s_{-1}\right\} & x & x\\ x & \left\{s_{0}\right\} & \left\{s_{1},s_{2},s_{2}\right\} & x & x\\ \left\{s_{1},s_{1},s_{1}\right\} & \left\{s_{-1},s_{-2},s_{-2}\right\} & \left\{s_{0}\right\} & \left\{s_{3},s_{3},s_{3}\right\} & \left\{s_{0},s_{1},s_{2}\right\}\\ x & x & \left\{s_{-3},s_{-3},s_{-3}\right\} & \left\{s_{0}\right\} & x\\ x & x & \left\{s_{0},s_{-1},s_{-2}\right\} & x & \left\{s_{0}\right\} \end{array}\right\}$$

$${\bar{B}}_{2}=\left\{\begin{array}{ccccc} \left\{s_{0}\right\} & \left\{s_{-1},s_{-1},s_{-1}\right\} & \left\{s_{0},s_{1},s_{1}\right\} & \left\{s_{1},s_{2},s_{3}\right\} & \left\{s_{2},s_{3},s_{3}\right\}\\ \left\{s_{1},s_{1},s_{1}\right\} & \left\{s_{0}\right\} & x & x & x\\ \left\{s_{0},s_{-1},s_{-1}\right\} & x & \left\{s_{0}\right\} & x & x\\ \left\{s_{-1},s_{-2},s_{-3}\right\} & x & x & \left\{s_{0}\right\} & x\\ \left\{s_{-2},s_{-3},s_{-3}\right\} & x & x & x & \left\{s_{0}\right\} \end{array}\right\}\;{\bar{B}}_{3}=\left\{\begin{array}{ccccc} \left\{s_{0}\right\} & \left\{s_{1},s_{2},s_{3}\right\} & \left\{s_{3},s_{4},s_{4}\right\} & \left\{s_{1},s_{2},s_{2}\right\} & x\\ \left\{s_{-1},s_{-2},s_{-3}\right\} & \left\{s_{0}\right\} & \left\{s_{1},s_{1},s_{1}\right\} & x & \left\{s_{-2},s_{-1},s_{-1}\right\}\\ \left\{s_{-3},s_{-4},s_{-4}\right\} & \left\{s_{-1},s_{-1},s_{-1}\right\} & \left\{s_{0}\right\} & x & \left\{s_{-2},s_{-2},s_{-2}\right\}\\ \left\{s_{-1},s_{-2},s_{-2}\right\} & x & x & \left\{s_{0}\right\} & \left\{s_{-1},s_{0},s_{0}\right\}\\ x & \left\{s_{2},s_{1},s_{1}\right\} & \left\{s_{2},s_{2},s_{2}\right\} & \left\{s_{1},s_{0},s_{0}\right\} & \left\{s_{0}\right\} \end{array}\right\}\;{\bar{B}}_{4}=\left\{\begin{array}{ccccc} \left\{s_{0}\right\} & \left\{s_{-2},s_{-2},s_{-2}\right\} & \left\{s_{-1},s_{-1},s_{-1}\right\} & x & \left\{s_{0},s_{1},s_{2}\right\}\\ \left\{s_{2},s_{2},s_{2}\right\} & \left\{s_{0}\right\} & \left\{s_{0},s_{1},s_{1}\right\} & x & \left\{s_{3},s_{4},s_{4}\right\}\\ \left\{s_{1},s_{1},s_{1}\right\} & \left\{s_{0},s_{-1},s_{-1}\right\} & \left\{s_{0}\right\} & x & \left\{s_{3},s_{3},s_{3}\right\}\\ x & x & x & \left\{s_{0}\right\} & x\\ \left\{s_{0},s_{-1},s_{-2}\right\} & \left\{s_{-3},s_{-4},s_{-4}\right\} & \left\{s_{-3},s_{-3},s_{-3}\right\} & x & \left\{s_{0}\right\} \end{array}\right\}$$

Obviously, ${\bar{B}}_{1}$ and ${\bar{B}}_{2}$ belong to the first category, ${\bar{B}}_{3}$ belongs to the second category, ${\bar{B}}_{4}$ belongs to the third category.

**Step 3.** Estimate the missing information in ${\bar{B}}_{1}$, ${\bar{B}}_{2}$, ${\bar{B}}_{3}$, and ${\bar{B}}_{4}$. The complete HFLPR ${\bar{B}}_{1}^{\prime }$ is obtained as

$${\bar{B}}_{1}^{\prime }=\left\{\begin{array}{ccccc} \left\{s_{0}\right\} & \left\{s_{-2},s_{-3},s_{-3}\right\} & \left\{s_{-1},s_{-1},s_{-1}\right\} & \left\{s_{2},s_{2},s_{2}\right\} & \left\{s_{-1},s_{0},s_{1}\right\}\\ \left\{s_{2},s_{3},s_{3}\right\} & \left\{s_{0}\right\} & \left\{s_{1},s_{2},s_{2}\right\} & \left\{s_{4},s_{5},s_{5}\right\} & \left\{s_{1},s_{3},s_{4}\right\}\\ \left\{s_{1},s_{1},s_{1}\right\} & \left\{s_{-1},s_{-2},s_{-2}\right\} & \left\{s_{0}\right\} & \left\{s_{3},s_{3},s_{3}\right\} & \left\{s_{0},s_{1},s_{2}\right\}\\ \left\{s_{-2},s_{-2},s_{-2}\right\} & \left\{s_{-4},s_{-5},s_{-5}\right\} & \left\{s_{-3},s_{-3},s_{-3}\right\} & \left\{s_{0}\right\} & \left\{s_{-3},s_{-2},s_{-1}\right\}\\ \left\{s_{1},s_{0},s_{-1}\right\} & \left\{s_{-1},s_{-3},s_{-4}\right\} & \left\{s_{0},s_{-1},s_{-2}\right\} & \left\{s_{3},s_{2},s_{1}\right\} & \left\{s_{0}\right\} \end{array}\right\}$$

Since ${\bar{B}}_{1}^{\prime }$ has linguistic terms that fall outside $\left\{s_{\alpha }|\alpha \in [-\tau ,\tau ]\right\}$, the transformation function F(x)=τx/(τ+a) is applied to construct a new HFLPR ${\bar{B}}_{1}^{\prime \prime }$ and a=5−4=1. Then, we have

$${\bar{B}}_{1}^{\prime \prime }=\left\{\begin{array}{ccccc} \left\{s_{0}\right\} & \left\{s_{-1.6},s_{-2.4},s_{-2.4}\right\} & \left\{s_{-0.8},s_{-0.8},s_{-0.8}\right\} & \left\{s_{1.6},s_{1.6},s_{1.6}\right\} & \left\{s_{-0.8},s_{0},s_{0.8}\right\}\\ \left\{s_{1.6},s_{2.4},s_{2.4}\right\} & \left\{s_{0}\right\} & \left\{s_{0.8},s_{1.6},s_{1.6}\right\} & \left\{s_{3.2},s_{4},s_{4}\right\} & \left\{s_{0.8},s_{2.4},s_{3.2}\right\}\\ \left\{s_{0.8},s_{0.8},s_{0.8}\right\} & \left\{s_{-0.8},s_{-1.6},s_{-1.6}\right\} & \left\{s_{0}\right\} & \left\{s_{2.4},s_{2.4},s_{2.4}\right\} & \left\{s_{0},s_{0.8},s_{1.6}\right\}\\ \left\{s_{-1.6},s_{-1.6},s_{-1.6}\right\} & \left\{s_{-3.2},s_{-4},s_{-4}\right\} & \left\{s_{-2.4},s_{-2.4},s_{-2.4}\right\} & \left\{s_{0}\right\} & \left\{s_{-2.4},s_{-1.6},s_{-0.8}\right\}\\ \left\{s_{0.8},s_{0},s_{-0.8}\right\} & \left\{s_{-0.8},s_{-2.4},s_{-3.2}\right\} & \left\{s_{0},s_{-0.8},s_{-1.6}\right\} & \left\{s_{2.4},s_{1.6},s_{0.8}\right\} & \left\{s_{0}\right\} \end{array}\right\}$$

Similarly, we can obtain

$${\bar{B}}_{2}^{\prime }=\left\{\begin{array}{ccccc} \left\{s_{0}\right\} & \left\{s_{-1},s_{-1},s_{-1}\right\} & \left\{s_{0},s_{1},s_{1}\right\} & \left\{s_{1},s_{2},s_{3}\right\} & \left\{s_{2},s_{3},s_{3}\right\}\\ \left\{s_{1},s_{1},s_{1}\right\} & \left\{s_{0}\right\} & \left\{s_{1},s_{2},s_{2}\right\} & \left\{s_{2},s_{3},s_{4}\right\} & \left\{s_{3},s_{4},s_{5}\right\}\\ \left\{s_{0},s_{-1},s_{-1}\right\} & \left\{s_{-1},s_{-2},s_{-2}\right\} & \left\{s_{0}\right\} & \left\{s_{1},s_{1},s_{2}\right\} & \left\{s_{2},s_{2},s_{2}\right\}\\ \left\{s_{-1},s_{-2},s_{-3}\right\} & \left\{s_{-2},s_{-3},s_{-4}\right\} & \left\{s_{-1},s_{-1},s_{-2}\right\} & \left\{s_{0}\right\} & \left\{s_{1},s_{1},s_{0}\right\}\\ \left\{s_{-2},s_{-3},s_{-3}\right\} & \left\{s_{-3},s_{-4},s_{-5}\right\} & \left\{s_{-2},s_{-2},s_{-2}\right\} & \left\{s_{-1},s_{-1},s_{0}\right\} & \left\{s_{0}\right\} \end{array}\right\}$$

$${\bar{B}}_{2}^{\prime \prime }=\left\{\begin{array}{ccccc} \left\{s_{0}\right\} & \left\{s_{-0.8},s_{-0.8},s_{-0.8}\right\} & \left\{s_{0},s_{0.8},s_{0.8}\right\} & \left\{s_{0.8},s_{1.6},s_{2.4}\right\} & \left\{s_{1.6},s_{2.4},s_{2.4}\right\}\\ \left\{s_{0.8},s_{0.8},s_{0.8}\right\} & \left\{s_{0}\right\} & \left\{s_{0.8},s_{1.6},s_{1.6}\right\} & \left\{s_{1.6},s_{2.4},s_{3.2}\right\} & \left\{s_{2.4},s_{3.2},s_{4}\right\}\\ \left\{s_{0},s_{-0.8},s_{-0.8}\right\} & \left\{s_{-0.8},s_{-1.6},s_{-1.6}\right\} & \left\{s_{0}\right\} & \left\{s_{0.8},s_{0.8},s_{1.6}\right\} & \left\{s_{1.6},s_{1.6},s_{1.6}\right\}\\ \left\{s_{-0.8},s_{-1.6},s_{-2.4}\right\} & \left\{s_{-1.6},s_{-2.4},s_{-3.2}\right\} & \left\{s_{-0.8},s_{-0.8},s_{-1.6}\right\} & \left\{s_{0}\right\} & \left\{s_{0.8},s_{0.8},s_{0}\right\}\\ \left\{s_{-1.6},s_{-2.4},s_{-2.4}\right\} & \left\{s_{-2.4},s_{-3.2},s_{-4}\right\} & \left\{s_{-1.6},s_{-1.6},s_{-1.6}\right\} & \left\{s_{-0.8},s_{-0.8},s_{0}\right\} & \left\{s_{0}\right\} \end{array}\right\}\;{\bar{B}}_{3}^{\prime }=\left\{\begin{array}{ccccc} \left\{s_{0}\right\} & \left\{s_{1},s_{2},s_{3}\right\} & \left\{s_{3},s_{4},s_{4}\right\} & \left\{s_{1},s_{2},s_{2}\right\} & \left\{s_{1},s_{-1},s_{-2}\right\}\\ \left\{s_{-1},s_{-2},s_{-3}\right\} & \left\{s_{0}\right\} & \left\{s_{1},s_{1},s_{1}\right\} & \left\{s_{0},s_{0},s_{-1}\right\} & \left\{s_{-2},s_{-1},s_{-1}\right\}\\ \left\{s_{-3},s_{-4},s_{-4}\right\} & \left\{s_{-1},s_{-1},s_{-1}\right\} & \left\{s_{0}\right\} & \left\{s_{-1.5},s_{-1.5},s_{-2}\right\} & \left\{s_{-2},s_{-2},s_{-2}\right\}\\ \left\{s_{-1},s_{-2},s_{-2}\right\} & \left\{s_{0},s_{0},s_{1}\right\} & \left\{s_{1.5},s_{1.5},s_{2}\right\} & \left\{s_{0}\right\} & \left\{s_{-1},s_{0},s_{0}\right\}\\ \left\{s_{-1},s_{1},s_{2}\right\} & \left\{s_{2},s_{1},s_{1}\right\} & \left\{s_{2},s_{2},s_{2}\right\} & \left\{s_{1},s_{0},s_{0}\right\} & \left\{s_{0}\right\} \end{array}\right\}$$

It is noted that no element falls outside in $\left\{s_{\alpha }|\alpha \in [-\tau ,\tau ]\right\}$. Thus, ${\bar{B}}_{3}^{\prime }={\bar{B}}_{3}^{\prime \prime }$.

To estimate the missing information in ${\bar{B}}_{4}$, the distance between ${\bar{B}}_{4}$ to ${\bar{B}}_{1}^{\prime \prime }$, ${\bar{B}}_{2}^{\prime \prime }$, and ${\bar{B}}_{3}^{\prime \prime }$ are first calculated, respectively, and then we can obtain $d\left({\bar{B}}_{1}^{\prime \prime },{\bar{B}}_{4}\right)=0.1517$, $d\left({\bar{B}}_{2}^{\prime \prime },{\bar{B}}_{4}\right)=0.1416$, $d\left({\bar{B}}_{3}^{\prime \prime },{\bar{B}}_{4}\right)=0.4930$. Given ζ=0.20, the missing elements of ${\bar{B}}_{4}$ are calculated as the arithmetic mean of the corresponding values in ${\bar{B}}_{1}^{\prime \prime }$ and ${\bar{B}}_{2}^{\prime \prime }$, and thus, we have

$${\bar{B}}_{4}^{\prime }={\bar{B}}_{4}^{\prime \prime }=\left\{\begin{array}{ccccc} \left\{s_{0}\right\} & \left\{s_{-2},s_{-2},s_{-2}\right\} & \left\{s_{-1},s_{-1},s_{-1}\right\} & \left\{s_{1.2},s_{1.6},s_{2}\right\} & \left\{s_{0},s_{1},s_{2}\right\}\\ \left\{s_{2},s_{2},s_{2}\right\} & \left\{s_{0}\right\} & \left\{s_{0},s_{1},s_{1}\right\} & \left\{s_{2},s_{3.2},s_{3.6}\right\} & \left\{s_{3},s_{4},s_{4}\right\}\\ \left\{s_{1},s_{1},s_{1}\right\} & \left\{s_{0},s_{-1},s_{-1}\right\} & \left\{s_{0}\right\} & \left\{s_{1.6},s_{1.6},s_{2}\right\} & \left\{s_{3},s_{3},s_{3}\right\}\\ \left\{s_{-1.2},s_{-1.6},s_{-2}\right\} & \left\{s_{-2},s_{-3.2},s_{-3.6}\right\} & \left\{s_{-1.6},s_{-1.6},s_{-2}\right\} & \left\{s_{0}\right\} & \left\{s_{-0.8},s_{-0.4},s_{-0.4}\right\}\\ \left\{s_{0},s_{-1},s_{-2}\right\} & \left\{s_{-3},s_{-4},s_{-4}\right\} & \left\{s_{-3},s_{-3},s_{-3}\right\} & \left\{s_{0.8},s_{0.4},s_{0.4}\right\} & \left\{s_{0}\right\} \end{array}\right\}$$

**Step 4.** By Equation (19) and Equation (20), we can obtain the objective weight of experts: $u={\left(0.19,0.19,0.33,0.29\right)}^{T}$ and the comprehensive weight of experts: $\omega ={\left(0.19,0.19,0.33,0.29\right)}^{T}$.

**Step 5.** Fuse all the individuals HFLPRs into a collective HFLPR:

$${\bar{B}}^{c}=\left\{\begin{array}{ccccc} \left\{s_{0}\right\} & \left\{s_{-0.71},s_{-0.53},s_{-0.19}\right\} & \left\{s_{0.59},s_{1.03},s_{1.03}\right\} & \left\{s_{1.13},s_{1.52},s_{2.00}\right\} & \left\{s_{0.48},s_{0.42},s_{0.53}\right\}\\ \left\{s_{0.71},s_{0.53},s_{0.19}\right\} & \left\{s_{0}\right\} & \left\{s_{0.63},s_{1.23},s_{1.23}\right\} & \left\{s_{1.45},s_{2.14},s_{2.08}\right\} & \left\{s_{0.82},s_{1.89},s_{2.20}\right\}\\ \left\{s_{-0.59},s_{-1.03},s_{-1.03}\right\} & \left\{s_{-0.63},s_{-1.23},s_{-1.23}\right\} & \left\{s_{0}\right\} & \left\{s_{0.58},s_{0.58},s_{0.68}\right\} & \left\{s_{0.51},s_{0.67},s_{0.82}\right\}\\ \left\{s_{-1.13},s_{-1.52},s_{-2.00}\right\} & \left\{s_{-1.45},s_{-2.14},s_{-2.08}\right\} & \left\{s_{-0.58},s_{-0.58},s_{-0.68}\right\} & \left\{s_{0}\right\} & \left\{s_{-0.87},s_{-0.27},s_{-0.27}\right\}\\ \left\{s_{-0.48},s_{-0.42},s_{-0.53}\right\} & \left\{s_{-0.82},s_{-1.89},s_{-2.20}\right\} & \left\{s_{-0.51},s_{-0.67},s_{-0.82}\right\} & \left\{s_{0.87},s_{0.27},s_{0.27}\right\} & \left\{s_{0}\right\} \end{array}\right\}$$

**Step 6.** The score values $s(b_{i}^{c})$(i=1,2,3,4,5) of the alternatives are obtained as $s(b_{1}^{c})=0.0764$, $s(b_{2}^{c})=0.1573$, $s(b_{3}^{c})=-0.0198$, $s(b_{4}^{c})=-0.1414$, and $s(b_{5}^{c})=-0.0722$. Therefore, the ranking of alternative is $x_{2}>x_{1}>x_{3}>x_{5}>x_{4}$. Thus, the best alternative is $x_{2}$.

### 6.2. Comparisons and Analyses

Some studies have been carried out to address the GDM problems with HFLPRs. A comprehensive comparison between the GDM model proposed in this paper and the existing models is provided.

(1) After defining the syntax, semantics and the context-free grammar related to HFLTSs, Rodríguez et al. [6] transformed the HFLPRs provided by experts into HFLTSs and obtained their envelopes to obtain the optimistic and pessimistic collective preference relations and establish the vector of alternatives. Then the non-dominance choice degree was used to rank alternatives and select the best one.
(2) Liu et al. [13] transformed the HFLPRs provided by experts into their corresponding 2-tuple fuzzy preference relations. Afterwards, a consistency reaching method to make the 2-tuple fuzzy preference relations acceptable was proposed. Finally, they utilized the linguistic 2-tuple arithmetic mean operator to rank the alternatives and obtain the best one.
(3) Wu and Xu [16] first introduced a consistency measure of HFLPR. If a HFLPR was not additive consistent, they developed an algorithm to improve the consistency degree. A consensus reaching model based on the distances between experts was introduced. Finally, the HFLWA operator was used to obtain the ranking of alternatives.
(4) Based on the envelope matrices of HFLPRs, Wang and Gong [19] constructed the equivalent interval-valued fuzzy preference relations and transformed them into the normal distributed preference relation. They ranked the alternatives and selected the best one based on the chance-restricted model.
(5) After calculating the weight vector of the experts, Gou et al. [24] calculated the synthetic HFLPR and the averaging value of each alternative over the other alternatives. They obtained a complementary matrix and summed all the elements in each line of it. Finally, the ranking of alternatives in descending order was obtained.
(6) Zhang and Wu [18] proposed a method to obtain the acceptable multiplicative consistent HFLPRs. Based on these, they used the HFLWA and hesitant fuzzy linguistic weighted geometric (HFLWG) operator to aggregate the preferences and rank the alternatives.

Table 1 lists the differences between our GDM model and the other GDM models mentioned above. The proposed method can deal with the problems with IHFLPRs, while the other methods can only solve the problems with complete HFLPRs. As we all know, the experts often cannot provide complete HFLPRs owing to various reasons, such as time restriction or workload. It is necessary for us to construct a model to obtain the best alternative from IHFLPRs. Both incomplete and complete HFLPRs can be solved by our proposed method.

Besides, there are also many other methods for flood disaster risk evaluation. Hall et al. [32] proposed a large scale regional flood risk assessment method based on the system reliability theory, and applied it to the assessment of future risk assessment in England and Wales. Apel et al. [33] combined the probability model and deterministic simulation to assess regional flood disaster risk. Utta et al. [34] systematically studied the direct economic losses assessment method by using an empirical formula. Including the proposed model in this paper, these methods belong to the simulation evaluation method. Owing to the fact that the flood control system is composed of many factors, the risk assessment must take multiple factors into consideration. This makes the routine evaluation usually care for one fact but lose the other. In particular, the real-time evaluation of flood disaster risk has obtained much attention from society. The key to real-time risk assessment is the faster extraction of information. Calculation efficiency should also be considered. However, there are few mature flood disaster risk assessment systems. Because real-time data cannot be accurate enough, the reliability and credibility of evaluative results cannot be guaranteed. As a result, the experiences and knowledge of experts are particularly important. Fast risk assessment under uncertainty is a significant advantage of our proposed GDM model.

## 7. Conclusions

In GDM, the experts often provide IHFLPRs with some missing information. In this paper, some properties of the additive consistent HFLPR were first discussed. Then the definition of the IHFLPR, the normalized IHFLPR, and the acceptable IHFLPR were introduced. Afterwards, three procedures to estimate the missing information in the IHFLPRs were proposed. The first one was used to deal with the situation in which the IHFLPR has only *n* − 1 judgments over all alternatives. The missing information of the IHFLPR with more than *n* − 1 known judgments was estimated by the second procedure. The third procedure was used to deal with ignorance situations. A method to determine the experts’ weights and a selection process to derive the optimal ranking from a HFLPR was developed. Based on this, an algorithm for GDM with IHFLPRs was established. A real-world case about flood disaster risk evaluation was used to verify the feasibility of our model. A comprehensive comparative analysis to justify the advantage of our model was also presented.

This study still has some deficiencies. For instance, there are some other methods that can be developed to estimate the missing information. Different methods may lead to different results, while this paper only uses the linear estimation method. This is a topic that deserves further discussion.

In the near future, we will study the consistency improving process for an unacceptable consistent IHFLPR and the consensus reaching method for IHFLPRs in GDM [35]. Given that the probabilistic linguistic term set [36] and the probabilistic linguistic preference relation [37] is a hot research topic, the GDM method with the incomplete probabilistic linguistic preference relations will be investigated.

## Figures and Tables

**Table 1:** Comparison between the proposed model and the other models.

References	Preference Relation	Method Used	Consistency Measure
Our method	Complete or Incomplete HFLPR	Additive consistency	No
Rodríguez et al. [6]	HFLPR	Envelope	No
Liu et al. [13]	HFLPR	Envelope	Additive
Wu and Xu [15]	HFLPR	Interactive	Yes
Wang and Gong [18]	HFLPR	Envelope	No
Gou et al. [23]	HFLPR	Compatibility	No
Zhang and Wu [17]	HFLPR	Normalization	Multiplicative
